# Genetic mapping of resistance to *Fusarium oxysporum* f. sp. *tulipae* in tulip

**DOI:** 10.1007/s11032-015-0316-3

**Published:** 2015-05-07

**Authors:** Nan Tang, Theo van der Lee, Arwa Shahin, Maarten Holdinga, Paul Bijman, Matteo Caser, Richard G. F. Visser, Jaap M. van Tuyl, Paul Arens

**Affiliations:** Wageningen UR Plant Breeding, Wageningen University and Research Centre, Droevendaalsesteeg 1, 6708 PB Wageningen, The Netherlands; Plateau Flower Research Centre, Qinghai University, Xining, 810016 Qinghai China; Biointeractions and Plant Health, Wageningen University and Research Centre, Droevendaalsesteeg 1, 6708 PB Wageningen, The Netherlands; Department of Agricultural, Forest and Food Sciences, University of Turin, Largo Paolo Braccini 2, 10095 Grugliasco, TO Italy; Department of Horticulture, Agriculture Faculty, Damascus University, Damascus, Syria

**Keywords:** *Tulipa*, Fungal disease, SNP, Genetic map, Green fluorescent protein (GFP), Quantitative trait

## Abstract

*Fusarium oxysporum* is a major problem in the production of tulip bulbs. Breeding for resistant cultivars through a conventional approach is a slow process due to the long life cycle of tulip. Until now, marker-assisted selection (MAS) has been hampered by the large genome size and the absence of a genetic map. This study is aimed at construction of the first genetic map for tulip and at the identification of loci associated with resistance to *F. oxysporum*. A cross-pollinated population of 125 individuals segregating for *Fusarium* resistance was obtained from *Tulipa gesneriana* “Kees Nelis” and *T. fosteriana* “Cantata.” *Fusarium* resistance of the mapping population was evaluated through a soil infection test in two consecutive years, and a spot inoculation test in which a green fluorescent protein tagged *Fusarium* strain was used for inoculation. The genetic maps have been constructed for the parents separately. The genetic map of “Kees Nelis” comprised 342 markers on 27 linkage groups covering 1707 cM, while the map of “Cantata” comprised 300 markers on 21 linkage groups covering 1201 cM. Median distance between markers was 3.9 cM for “Kees Nelis” and 3.1 cM for “Cantata.” Six putative quantitative trait loci (QTLs) for *Fusarium* resistance were identified, derived from both parents. QTL2, QTL3, and QTL6 were significant in all disease tests. For the flanking markers of the QTLs, phenotypic means of the two allelic groups, segregating from a parent for such a marker, were significantly different. These markers will be useful for the development of MAS in tulip breeding.

**Electronic supplementary material:**

The online version of this article (doi:10.1007/s11032-015-0316-3) contains supplementary material, which is available to authorized users.

## Introduction

Tulip, belonging to the genus *Tulipa* L. in the family Liliaceae, is one of the most important ornamental crops in the world. The genus consists of more than 100 species and thousands of derived cultivars (Botschantzeva 1982). The showy tulip flowers are popular in parks, gardens, or used as cut flowers and potted plants.

*Fusarium oxysporum* f.sp. *tulipae* causes the most serious fungal disease in tulips called “Bulb rot” or “Basal rot.” The disease is widespread and occurs primarily during storage. *Fusarium* produces dark brown spots on the top or side of tulip bulbs and further causes bulb base or root rot. When diseased bulbs are forced in the greenhouse for flowering, stunted growth and leaf yellowing will occur within a few weeks, and plants generally die before flowering. In addition to the direct symptoms caused by *Fusarium*, the fungus also produces large quantities of ethylene (Gerardo et al. 2007). High concentrations of ethylene are a serious problem in tulip bulbs in storage as it can increase respiration of bulbs, reduce shoot and root elongation, and subsequently increase flower bud abortion (Cerveny and Miller 2010; Kanneworff and van der Plas 1994). In addition, it has been suggested that ethylene represses the defense ability of the host plant since it prevents the synthesis of antifungal compounds such as tulipaline (Miller et al. 2004). Another unwanted symptom is gummosis. Gum inside tulip bulbs fills up the spaces between bulb scales and forms blisters when a large amount of gum is produced. The presence of gummosis indicates that tulip bulbs have been exposed to ethylene. Compared to other tested formae speciales, *Fusarium oxysporum* f.sp. *tulipae* ethylene production is at least 2000 times higher (Kamerbeek 1975).

Agronomic measures such as avoidance of wounding, removal of diseased bulbs, and crop rotation are insufficient to control the disease, and therefore, control strategies rely on the frequent use of chemicals. Since *Fusarium* can survive in the soil for more than 6 year, a continuous use of chemicals is required once a field is contaminated by *Fusarium*. Prolonged use of chemicals creates an environmental risk and is likely to lead to fungicide resistance in the pathogen.

Breeding for *Fusarium*-resistant tulip cultivars is therefore an attractive alternative. Various cultivars were screened in previous studies (van Eijk et al. 1978), and some cultivars in *Tulipa gesneriana* were found resistant to *Fusarium*. These cultivars were further used as parents of crossings to make new hybrids. Unfortunately, conventional breeding is a very slow process. Tulip has a long life cycle (4–5 years), and it takes a long time to obtain new cultivars for desirable traits and to propagate bulbs for commercial use (van Tuyl and van Creij 2006). Therefore, marker-assisted selection (MAS) has the potential to speed up the breeding process and to increase efficiency. MAS is already used in many crops such as cotton, rice, maize, potato, and tomato (Zhang et al. 2003; Dokku et al. 2013; Foolad and Panthee 2013; Li et al. 2013; Yang et al. 2013). The obstacles to carry out MAS in tulip breeding at present are that tulip has a large genome (1C ≈ 30 GB), only few molecular markers have been published (Shahin et al. 2012), and no genetic map is available. The retrieval of single nucleotide polymorphisms (SNPs) can promote linkage analysis for tulip (Shahin et al. 2012).

Apart from the need for a high-quality genetic map, the resistance of tulip to *F. oxysporum* is poorly understood. *F. oxysporum* attacks more than a hundred different hosts, and in some plant pathosystems, a clear gene-for-gene resistance was found, e.g., in tomato (Sarfatti et al. 1989; Scott et al. 2004; Shahin and Spivey 1986), cucumber (Netzer et al. 1977), West Indian Gherkin (*Cucumis anguria* L.) (Matsumoto and Miyagi 2012), and *Arabidopsis* (Diener and Ausubel 2005). However, in other plant pathosystems, a quantitative response was observed, for example, *Fusarium* head blight in barley and wheat (de la Pena et al. 1999; Gervais et al. 2003), *Fusarium* root rot in common bean (Roman-Aviles and Kelly 2005), and *Fusarium* wilt in flax (Spielmeyer et al. 1998). In this latter case, accurate quantitative phenotyping is a prerequisite in order to identify QTLs.

Previous studies evaluated *Fusarium* resistance in tulip cultivars and breeding lines during a whole growing season and used the percentage of healthy bulbs as the criteria for the degree of resistance (van Eijk et al. 1978, 1979). This traditional visual scoring approach was slow but efficient in screening resistant plants, but quicker phenotyping is needed to screen larger numbers of genotypes in breeding, and more accurate phenotype data are needed in order to precisely identify QTLs. Using green fluorescent protein (GFP)-tagged pathogens, the infection process can be monitored in detail and quantitatively. By using GFP-transformed fungal strains, the pathogenic and nonpathogenic lifestyles in *Colletotrichum acutatum* were unravelled (Horowitz et al. 2002). A GFP-transformed strain of *Fusarium oxysporum* f.sp. *radicis*-*lycopersici* was efficiently used in studying its colonization and infection process in tomato (Lagopodi et al. 2002). Assaying of fluorescence signal from a GFP-transformed fungus is an accurate, fast and easy approach to quantify the growth of fungi inside host plants (Chen et al. 2003; Li et al. 2011).

The aims of this study were (1) to construct the first genetic linkage maps for tulip, using a combined set of single nucleotide polymorphism, amplified fragment length polymorphism (AFLP), nucleotide-binding site (NBS), and simple sequence repeat (SSR) markers; (2) to evaluate *Fusarium* resistance tests for this mapping population by a fast visual scoring approach and by a GFP imaging approach; and 3) to perform QTL analysis and identify putative QTLs which can be further used for promoting marker-assisted breeding of tulip.

## Materials and methods

### Plant material

A mapping population consisting of 125 F_1_ progeny was derived from a cross between *Tulipa gesneriana* L. “Kees Nelis” × *T. fosteriana* L. “Cantata.” The parents “Kees Nelis” (KN) and “Cantata” (CA) were cultivars introduced in 1951 and 1941, and differ in their level of *Fusarium* resistance. “Kees Nelis” is more resistant to *F. oxysporum,* while “Cantata” is more susceptible. The mapping population of 125 offspring has been created in 1989, and plants have been vegetatively propagated ever since.

### Determination of the level of *Fusarium* resistance in tulip bulbs

Two methods to determine *Fusarium* resistance were used: a spot inoculation test and a soil infection test. In the spot inoculation test, bulb skin was peeled off, and bulbs were inoculated by *Fusarium* strains that have been transformed with the GFP gene, and infection was quantified by measuring the amount of GFP signal using an imaging system. In the soil infection, bulbs were incubated in *Fusarium*-infected soil, and infection was visually evaluated. *Fusarium* resistance of the parents and progenies, as well as that of four cultivars (‘Ile de France’, ‘Bellona’, ‘Christmas Dream’ and ‘Monte Carlo’) with known differences in resistance that were used as reference and indicator (disease progress) genotypes, was evaluated.

### Spot inoculation test using a GFP-tagged *Fusarium* strain

To quantify the resistance level against *Fusarium*, a new phenotyping platform developed at Plant Research International (PRI), Wageningen UR, was applied. Three *Fusarium* strains (Tu47, Tu58, and Tu67 obtained from PPO Flowerbulbs, Wageningen UR, the Netherlands) were transformed with green fluorescent protein gene as described by Zhang et al. (2008). Before carrying out the resistance evaluation across the mapping population, the aggressiveness of each single strain was tested on the parents and reference cultivars. The most aggressive strain Tu67 was selected for testing the mapping population. Aside from the single-strain test, effects of combinations of different strains were tested on parents and reference cultivars.

For the inoculation, each tulip bulb was wounded by three 2-mm-deep punctures (each punch spaced 5 mm from the other two punches) after which bulbs were inoculated by gently dipping the bulbs on a cushion soaked with a GFP-tagged *Fusarium* suspension of 5 × 10^5^ spores per ml Mock solution (solution without *Fusarium*), and wild-type strain was inoculated as controls in the same way. Forty bulbs can be placed in the customized box fitting the imaging platform. Ten genotypes were placed per box with four replicate bulbs for each genotype (offspring, parents, and reference cultivars). For each genotype, in another box, four bulbs were placed as replicates. Bulbs of each genotype were placed in one column. Inoculated bulbs were incubated at >90 % RH and 24 °C for 14 days. Infection area (IA, percentage of infected area) was quantified using the *Fusarium* Screen Analysis Software. Obtained IA scores were analyzed in three steps. Firstly, outliers were detected according to the interquartile range rule (Tukey 1977). Outliers were excluded from calculation of the average score for each genotype per box. Secondly, the correlation between replicate bulbs in the two boxes was calculated. Individuals showing large variation between two boxes (IA of the second box was more than two times smaller or larger than the first box) were excluded from further analyses.

### Soil infection test

Compared with previous studies (van Eijk et al. 1978, 1979), resistance to *Fusarium* was tested using a modified approach in which assessments are made in a relative short time during the normal storage period of bulbs. Three *Fusariumoxysporum* strains (Tu47, Tu58, and Tu67) were grown on oatmeal separately and well mixed with soil substrate before use. Healthy bulbs with a diameter of 10–12 cm were selected for disease testing. Similar to the spot inoculation test, offspring, parents, and reference cultivars were tested. Each bulb was put into a separate mesh bag with 100 cl of the *Fusarium*-infected substrate, placed in a crate, and covered with moist vermiculite. All bulbs were randomized over crates. Plastic bags, with holes to prevent accumulation of ethylene, were used to wrap the crates so as to preserve moisture. All bulbs were kept at 20–24 °C for 8 weeks. Disease infection degree was scored on a 1–5 scale: 1 = healthy (clean and hard bulb), 2 = slightly infected (infection ≤10 %), 3 = moderately infected (10 % <infection ≤50 %), 4 = heavily infected (infection >50 %, still some hard bulb material left), and 5 = completely rotted. For each genotype, outliers of replicate bulbs were removed, and average score was calculated to represent the resistance level. The test was carried out in the last part of the normal bulb storage period (July–November) in two consecutive years (2011 and 2012). For each progeny genotype, five replicate bulbs were tested in each year. For the parents and reference cultivars (“Bellona,” “Christmas Dream,” “Ile de France,” and “Monte Carlo”), thirty bulbs each were tested.

### Assessment of skin quality

Skin quality was assessed in 2011 after the bulbs were harvested, and scoring the mapping population was performed on a 1–5 scale: 1 = very good (skin is intact), 2 = good (small fissures), 3 = moderate (a few cracks on the skin), 4 = poor (part of the skin fell off), and 5 = very poor (skin completely fell off).

### SNP genotyping

SNP markers were developed based on SNPs in expressed sequence tags as described by Shahin et al. (2012), which have been subsequently validated in a random set of cultivars (Tang et al. 2013). Marker names were based on the parent (KN or CA) showing the SNP variation followed by the contig number of the Tulip_All assembly as described by Shahin et al. (2012). SNP markers were used to genotype the parents and the mapping population using KASPar technology (LGC Genomics, http://www.lgcgenomics.com/). Genotype data were visualized by SNPViewer2 (LGC Genomics http://www.lgcgenomics.com/) and were filtered manually. Firstly, monomorphic markers and markers that showed no calls for both parents were excluded. Secondly, the segregation of each marker over the progeny was assessed. The expected segregation ratio for a SNP marker that is polymorphic in only one parent is 1:1, while segregation for SNP markers polymorphic in both parents is 1:2:1. Markers with strange segregation patterns were carefully checked, and explainable aberrant segregations (by assuming one/two null allele(s) in one or both parents) were rescored manually. Rescored SNPs were marked with a “C” at the end of the marker name, and their reliability was tested later in the linkage analysis. SNP markers showing unexplainable segregation patterns were discarded.

### SSR markers

EST-SSRs have been identified previously (Shahin et al. 2012). For marker use, SSRs were selected according to their repeat length: at least 10 repeats for dinucleotide motifs; seven repeats for trinucleotide motifs; and five repeats for tetra-, penta-, and hexanucleotide motifs. For compound SSRs, at least six repeat units were required. A total of 56 SSRs were selected, of which 25 from EST-contigs containing both ‘Kees Nelis’ and ‘Cantata’ reads, 19 from contigs with only ‘Kees Nelis’ reads and 12 from contigs with only ‘Cantata’ reads. Primers were ordered from Biolegio BV (Nijmegen, the Netherlands). Polymorphism of markers was first tested in the parents and 10 offspring. Polymorphic SSRs were selected, and PCR conditions were optimized (Table S1). The parents and offspring were genotyped using selected polymorphic SSRs on a Li-Cor 4300 DNA analyzer (LI-COR Corporate, Nebraska, USA). Genotype data were scored based on marker segregation type (Table S2).

### AFLP and NBS markers

AFLP markers were obtained according to Vos et al. (1995) with some slight modifications (see van Heusden et al. (2002)). In short, AFLP reactions were carried out with two different restriction enzyme combinations: seven *EcoRI/MseI* (E35M52T, E36M52G, E36M52T, E37M52G, E37M52T, E37M52, and E40M52A) and five *PstI/MseΙ* (P31M47, P31M48, P31M49, P31M50, and P31M54). The primer sequences have been described in detail by van Heusden et al. (2000). Six selective nucleotides were used for the two final primers in the combination *PstI/MseΙ* (+3, +3) and seven selective nucleotides for *EcoRI/MseI* (+3, +4). Amplified fragments were separated on denaturing polyacrylamide gels. NBS profiling (NBS2 and NBS6) was performed as described by Shahin et al. (2011) however using a Li-Cor 4300 DNA analyzer for the detection of fragments (see Caser et al. 2010). AFLP and NBS fragments were scored as present (1)/absent (0). All markers were scored twice, and inconsistent markers were checked. AFLP markers were named after the names of the primer combination, followed by a number representing the fragment position on the gel. Similarly, NBS markers were named according to the NBS primer used and a following number based on order of recognition.

### Genetic linkage map construction

Genetic linkage maps were constructed based on SNP, AFLP, NBS, and SSR markers using JoinMap 4.1 (van Ooijen 2011). Goodness of fit between observed and expected segregation ratios was tested using Chi-square testing. Highly skewed markers (*P* < 0.005) were excluded from linkage analysis. Parental linkage maps were constructed separately using a double pseudo-testcross strategy (Grattapaglia and Sederoff 1994).

Markers were grouped using the regression method at a minimum LOD threshold of 5. The Kosambi mapping function was used to convert recombination frequency to map distances in centi Morgan (cM). At first, a frame map was built using only SNP markers. AFLP, NBS, and SSR markers were later added. In all linkage analysis, identical loci were excluded before calculating linkage groups. Marker order was checked against the order of SNP markers in the frame map. The contribution of each marker to the average goodness-of-fit (mean Chi-square) and the nearest-neighbor fit (N.N. Fit) value was checked to confirm its most likely position in each linkage group. Markers showing large Chi-square contributions and causing suspect linkages were rechecked and discarded, if no clear misscores could be detected, to improve the map fit. Graphical genotyping was performed to visualize the map quality by identifying possible double recombinant events. For each linkage group, clustered AFLP markers which have the same phase and identical genotype scores across the population except for a few missing values were considered as duplicates and were reduced to one marker (retaining marker with no or fewest missing values).

Genome length was estimated using the method-of-moment estimator, *E*(*G*) = 2*MX/K* (Hulbert et al. 1988), where *M* is the number of informative meiosis, *X* is the map distance between two markers for which the expected LOD score is 3. *K* is the number of pairs of loci within the distance *X*, with a LOD value of at least 3.0. Since we analyzed the parents separately, only informative markers were analyzed, *M* = *n*(*n* − 1)/2, where n is the number of loci analyzed.

### QTL analysis

The means of visual scoring in both years and the IA mean of the GFP test were each used for QTL analysis separately. QTLs were detected using MapQTL6 (van Ooijen 2009) in either the KN map or the CA map. A threshold of *P* < 0.05 was set to identify markers significantly associated with *Fusarium* resistance by a nonparametric Kruskal–Wallis test. Based on the linkage map, interval mapping (IM) was performed to confirm the putative QTLs detected by Kruskal–Wallis. A genome-wide LOD threshold with a *P* value of 0.05 was calculated by a permutation test with 1000 replicates (van Ooijen 1999). Based on the result of interval mapping, MQM (multiple QTL models) was performed with the maximum likelihood mixture model using the closest markers as cofactors. Overall genotypic variation explained was estimated by ANOVA in Genstat 15th, and the phenotypic variation explained by a QTL was estimated in the IM procedure of MapQTL.

## Results

### Soil infection test

**Fig. 1:**
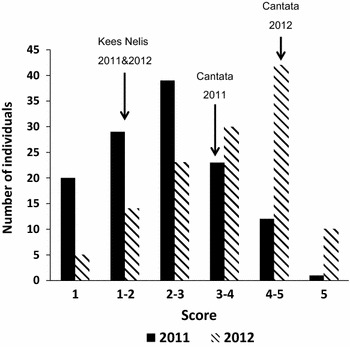
Distribution of the infection score of the soil infection test in parents *T. gesneriana* “Kees Nelis” (KN) and *T. fosteriana* “Cantata” (CA) and offspring in 2011 and 2012

Visual assessment of *Fusarium* resistance was carried out in two consecutive years (2011 and 2012). In 2011, the average score of parents “Kees Nelis” and “Cantata” was 1.3 (±0.6) and 3.5 (±1.5), respectively. The mean scores of reference cultivars “Bellona,” “Christmas Dream,” “Ile de France,” and “Monte Carlo” were 2.8 (±1.4), 1.6 (±1.0), 1.1 (±0.2), and 3.0 (±1.3). Mean scores of individuals in the mapping population ranged between 1.0 and 5.0, showing a continuous distribution from resistant to susceptible phenotypes (Fig. 1). Twenty progenies showed a high level of resistance, i.e., no infection in all replicate bulbs (score = 1), whereas thirteen progenies showed an average score higher than 4, indicating they were more susceptible than parent “Cantata.” In 2012, the severity of infection in general was higher than in 2011 with higher mean values for parents “Kees Nelis” (1.4 ± 0.7) and “Cantata” (4.4 ± 1.1) and for reference cultivars “Bellona,” “Christmas Dream,” “Ile de France,” and “Monte Carlo” of 2.7 (±0.9), 2.1(±1.3), 1.3 (±1.0), and 4.2 (±1.4), respectively. In line, fifty-two individuals of the progeny showed heavy infection (mean score higher than 4). Only five progenies showed a high level of resistance (score = 1). Transgressive segregation was observed in both years. In 2011, 19.2 % of the progenies showed to be more resistant than parent KN, while 16.0 % were more susceptible than CA. In 2012, 6.4 % of the progeny were more resistant than KN and 26.4 % were more susceptible than CA. The correlation of scores between 2011 and 2012 is 0.481 (*P* < 0.001).

### Spot inoculation test using GFP-tagged *Fusarium* strains

Parent “Cantata” was highly infected (infection area IA = 37.6 %) by *F. oxysporum* f.sp. *tulipae* isolate Tu67, while parent “Kees Nelis” showed high resistance, i.e., low infection (IA = 1.96 %). A total of 992 bulbs were screened for resistance scores from a total of 124 offspring genotypes. The replicate bulbs for each genotype that were placed in the same box usually showed a very similar infection level. However, 21 outliers in replicate 1 and 17 outliers in replicate 2 were detected. In most of these cases, all but one bulb showed a high infection level. Therefore, these outliers were regarded as failed inoculations and removed from the data set. A Pearson’s correlation coefficient of 0.648 between replicate boxes was found after removing the outliers. Eight offspring genotypes showed an extremely large variation between their replicate boxes. For example, progeny 89191-25 showed an average infection area of 41.1 % in box 1, but only 4.9 % in box 2. Averaging the values of such replicates may not reflect the real resistance level. These eight progenies were therefore discarded which led to an increase in the correlation coefficient of the replicate boxes to 0.730. IA of the mapping population showed a continuous distribution. Transgressive segregation was clearly observed. A total of 39 % of the progeny showed higher infection than susceptible parent CA, while 5.3 % of the progeny were more resistant than parent KN.

In general, the three disease tests in this study (two soil infection and one spot inoculation) showed moderate correlations. Correlation between the two soil infection tests was 0.48. Soil infection 2011 and spot inoculation were relative weakly correlated (*r* = 0.30), while correlation between soil infection 2012 and spot inoculation was stronger (*r* = 0.53).

### SNP genotyping

A total of 316 SNP markers (151 from KN, 165 from CA) were genotyped across the mapping population. Out of these SNP markers, 275 (88.6 %) segregated in the progeny (Table S3). In total, 122 SNP markers derived from “Kees Nelis” (KN_SNP) and 121 from “Cantata” (CA_SNP) segregated in that parent (<AB × AA> or <AA × AB>) only (Fig. S1A). Just for 1 KN_SNP and 4 CA_SNPs, segregation in both parents (<AB × AB>) was found (Fig. S1B). Twenty-seven SNP markers (11 KN_SNP, 16 CA_SNP) were discarded due to non-Mendelian segregations. For other SNP markers with a segregation deviating from normal expectations, the observed segregation could be explained by the presence of null allele(s) (Ø), and they were rescored manually. For example, in marker KN_24675, parent “Kees Nelis” is AB and “Cantata” is BB (Fig. S1C). Theoretically, the progeny for this marker will show two clusters AB and BB. However, also an “AA” cluster was present. Apparently, there is a null allele present in “Cantata” (BØ). The parental marker genotypes AB x BØ result in four allelic combinations with equal segregation ratios (AB, AØ, BB, BØ) in which AØ and BØ are visualized as homozygous AA and BB, respectively. Therefore, three groups (AA, AB, and BB) with a segregation of 1:1:2 were observed. This type of marker is a fully informative biallelic marker from one parent’s side and a partial informative marker from the other parent (one with the null allele: only for AB and AØ offspring genotypes). Consequently, this type of SNP marker can be converted and used in mapping which was done for 20 SNP markers (12 KN_SNP, 8 CA_SNP). The presence of two null alleles can be supposed when one parent shows a “no call” in replicate samples. For example, parent “Kees Nelis” showed a “no call” in both replicate samples for SNP marker CA_11914, whereas parent “Cantata” was heterozygous AB (Fig. S1D). Genotyping the offspring with such a marker (<ØØ × AB>) will result in two clusters AØ and BØ, which are visualized as AA and BB. From the heterozygous parent, such markers can be used as a biallelic marker and used as <AA × AB> in JoinMap. By doing so, 10 SNP markers (6 KN_SNP, 4 CA_SNP) that showed homozygous null alleles in one parent could be added for mapping.

### Genetic map

**Fig. 2:**
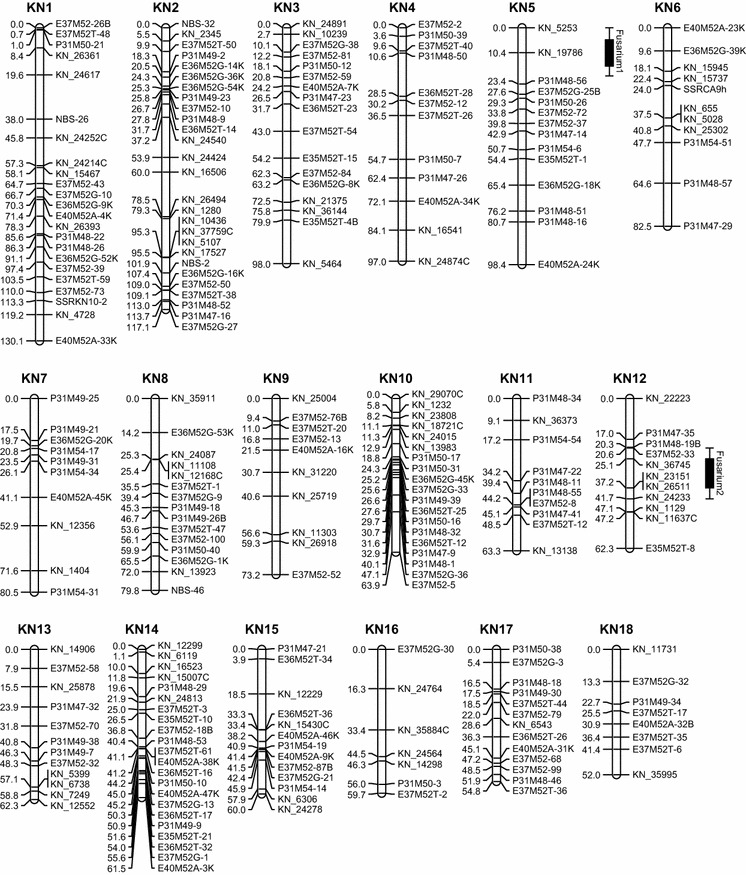

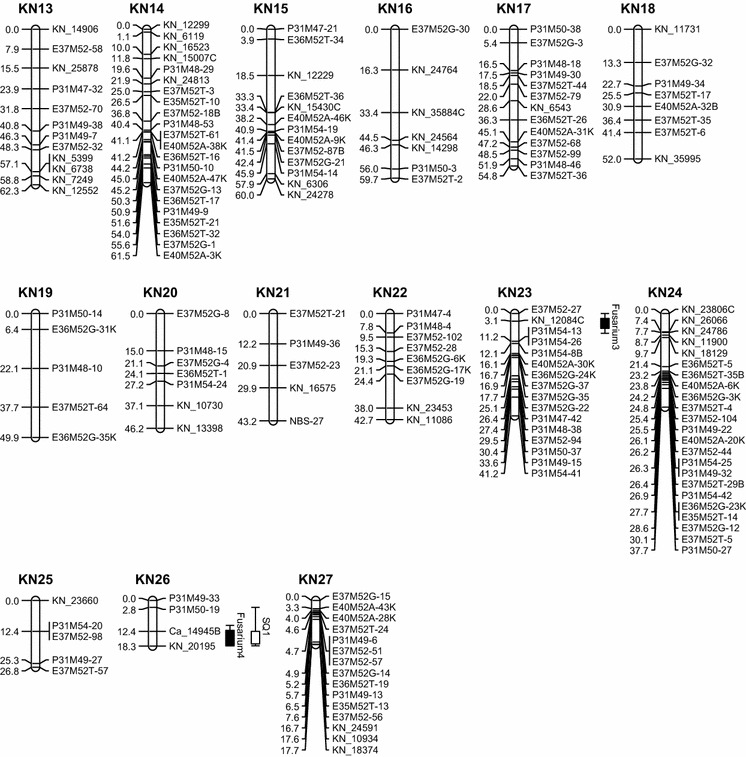

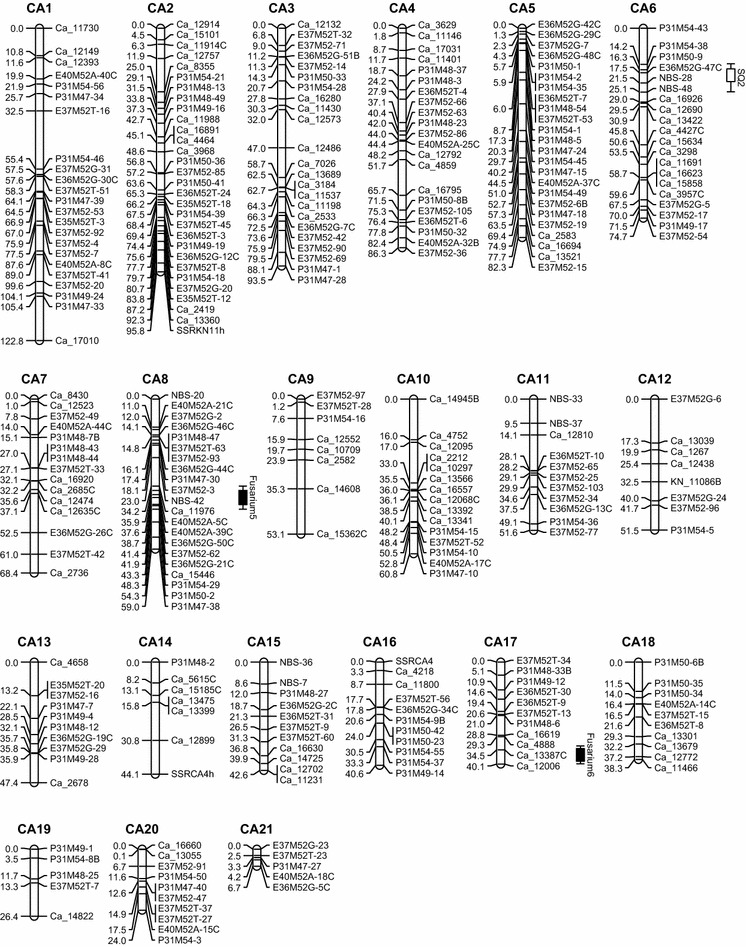
Genetic maps of *T. gesneriana* “Kees Nelis” and *T. fosteriana* “Cantata.” “KN” and “CA” represent linkage groups of “Kees Nelis” and “Cantata,” respectively. Putative QTLs were represented by *boxes* extended by lines representing the confidence intervals

Parental maps were obtained separately. For the maternal map (KN map), a total of 519 markers comprising 127 SNP (123 KN_SNP and 4 CA_SNP), 359 AFLP, 28 NBS, and 5 SSR markers were available for linkage mapping (Table 1). Seventy-five markers were excluded due to skewed segregation (*P* = 0.005). The remaining 444 markers (110 SNPs consist of 108 KN_SNP and 2 CA_SNP, 306 AFLPs, 25 NBSs, and 3 SSRs) were used for map construction, in which 392 segregated 1:1 (including 21 rescored KN_SNP markers). A total of 328 (83.7 %) markers which segregated in a 1:1 fashion were mapped, including 16 rescored markers. Fifty-two markers segregated 1:2:1 of which only 14 (26.9 %) were successfully mapped. In total, 342 markers were mapped in the KN map, while 102 (23.0 %) markers remained unmapped. The KN map was comprised of 27 linkage groups (Fig. 2) and covered 1707 cM (57 %) of the expected genome length of 2995 cM based on the method-of-moment estimator (Hulbert et al. 1988). Median distance between markers was 3.9 cM, and the largest distance was 18.8 cM (LG KN7). The length of the linkage groups ranged from 17.7 cM (LG KN27) to 130.1 cM (LG KN1). Average mean Chi-square of linkage groups was 1.11, ranging from 0.135 (LG26) to 3.708 (LG21).

**Table 1:** Summary of all markers (SNP, AFLP, NBS, and SSR) used for map construction

	KN	CA
Total number of polymorphic marker	519	438
Highly skewed marker	75	58
No. of markers used for map construction	444 (86 %)	380 (87 %)
No. of markers that segregated 1:1 or 3:1	392	328
No. of segregating markers mapped in each map 1:1/3:1	328	289
No. of markers that segregated 1:2:1	52	52
No. of segregating markers that mapped in each map 1:2:1	14	11

A total of 438 markers were polymorphic in parent CA, comprising 126 SNP (including 1 KN_SNP which segregated in 1:2:1), 287 AFLP, 18 NBS, and 7 SSR markers. Chi-square tests showed that 58 markers had a skewed segregation (*P* = 0.005). These highly skewed markers were excluded, and 380 markers were selected for the construction of the CA map, which comprised 108 SNP (107 CA_SNP and 1 KN_SNP), 252 AFLP, 14 NBS, and 6 SSR markers. Of the selected markers, 328 segregated in a 1:1 ratio, including 13 rescored SNP markers. A total of 289 (88.1 %) markers, including 10 rescored SNP markers, segregated in a 1:1 ratio and were successfully mapped on the CA map. The other 52 markers segregated in a 1:2:1 ratio, and 11 (21.2 %) of them were mapped. In the end, a total of 300 markers (99 SNP, 190 AFLP, 8 NBS, and 3 SSR markers) were mapped on 21 linkage groups (Fig. 2), while 80 (21.1 %) markers remained unmapped. The total length of the CA map was 1201 cM. The estimated genome size of the CA map was 2390 cM so the obtained genetic map covered about 50.3 % of the expected genome length. Median distance between markers was 3.1 cM, and the largest distance was 19.6 cM (LG CA2). The length of the linkage groups ranged from 6.7 cM (LG CA21) to 122.8 cM (LG CA1). Average mean Chi-square of the linkage groups was 1.19, ranging from 0.122 (LG CA16) to 1.967 (LG CA9).

Map integration between parental maps is possible if at least two bridge markers per linkage group are mapped. In this study, a total of 52 cosegregating markers were available for map construction. However, 49 of these were AFLP markers, and most of them remained either ungrouped or had to be excluded. This is due to the fact that AFLPs are dominant markers and as such have low information content and suffer from unsuccessful linkage phase determination. Only four bridge markers (of which two out of the three SNP bridge markers) could be mapped in both maps. E40M52A-32B was mapped in linkage group KN18 and CA4. KN_11086B was mapped in KN22 and CA12. P31M54-8B was mapped in KN23 and CA19. Ca_14945B was mapped in KN26 and CA10. These low numbers of bridge markers do not allow the calculation of integrated linkage maps. Numbering of linkage groups has been based on map length.

### QTL analysis

Six putative QTLs for *Fusarium* resistance were identified in the nonparametric Kruskal–Wallis test (Table 2). QTLs Fusarium2, Fusarium3, and Fusarium6 were found significant in all three disease tests. The significance level of QTLs varied in each of the three disease test. Fusarium2 was highly significant (*P* < 0.0001) in Visual2011 and Visual2012, whereas in the GFP test, it was only significant at level *P* < 0.05. Fusarium3 was highly significant in the Visual2011 (*P* < 0.0005) and GFP test (*P* < 0.005), while significant at *P* = 0.05 in Visual2012. Fusarium6 showed high significance (*P* < 0.001) in all three tests. QTLs Fusarium1, Fusarium4, and Fusarium5 were only found in the GFP test, and they were highly significant (*P* < 0.001). Subsequently, interval mapping (IM mapping) was used to identify putative QTLs at a 5 % genome-wide threshold significance level. The LOD scores of the QTLs were not high, and not all putative QTLs from the Kruskal–Wallis test were above the genome-wide threshold in IM mapping (Table 2). Fusarium1, Fusarium2, Fusarium3, and Fusarium4 were detected in the KN map. Although Fusarium1 showed high significance in the Kruskal–Wallis test, in IM mapping the LOD score (3.2) remained below the threshold (3.4). QTL Fusarium2 showed a LOD value of 4.4 in Visual2012 which explained 18.5 % of the variation. For Fusarium3, the highest LOD was observed in the Visual2011 (LOD = 3.4, genome-wide threshold = 3.3), and it explained 14.9 % of the variation. This is in agreement with the result of the Kruskal–Wallis test in which Visual2011 showed the highest significance among the three tests. LOD values of Fusarium3 detected in Visual2012 (2.8) and GFP (2.1) were relatively low. Fusarium4 was detected in the GFP test with a LOD score of 3.4, explaining 20.7 % of the total variance. QTLs Fusarium5 and Fusarium6 were found in the CA map. Fusarium5 was only detected in the GFP test with a high LOD value (4.3), explaining 16.0 % of the variance. Fusarium6 was only significant in Visual2012, explaining 13.6 % of the variation. The closest markers flanking the putative QTLs were selected as cofactors in MQM. The QTLs identified in interval mapping were still present in MQM. No extra minor QTL was revealed taking over the role of the nearby QTLs. The proportion of genotype variance explained in Visual2011, Visual2012, and GFP tests was 41.0, 60.8, and 59.9 %, respectively.

**Table 2:** QTLs for *Fusarium* resistance identified in different disease tests

QTL	LG	Flanking loci		Assay	Sig	GW	LOD peak	%Exp
Fusarium1	KN5	KN_19786	KN_5253	GFP	******	3.4	3.2	12.5
Fusarium2	KN12	KN_36745	KN_23151	Visual2011	*******	3.3	1.7	7.1
				Visual2012	*******	3.5	4.4	18.5
				GFP	**	3.4	2.4	14.5
Fusarium3	KN23	KN_12084C	P31M54-26	Visual2011	******	3.3	3.4	14.9
				Visual2012	**	3.5	2.8	12.4
				GFP	****	3.4	2.1	8.4
Fusarium4	KN26	Ca_14945B	KN_20195	GFP	*****	3.4	3.4	20.7
Fusarium5	CA8	Ca_11976	Ca_15446	GFP	******	2.9	4.3	16.0
Fusarium6	CA17	Ca_13387C	Ca_12006	Visual2011	******	3.2	3.1	12.2
				Visual2012	******	3.2	3.4	13.6
				GFP	*****	2.9	2.7	10.7

*LG* linkage group, *Sig* significance level of the QTL in Kruskal–Wallis test (*, **, ***, ****, *****, ****** refer to significant at *P* = 0.1, 0.05, 0.01, 0.005, 0.001, and 0.0005, respectively), *GW* genome-wide significant threshold level *P* < 0.05; %Exp: percentage explained variance by the QTL

Two QTLs for skin quality (SQ1 and SQ2) were identified (Fig. 2) using the same approach as used for the *Fusarium* resistance tests. SQ1 located on the KN map LG KN26 with a LOD of 2.34 and explained 12.6 % of the variation. SQ2 located on CA map LG CA6 with a LOD value of 4.07 and explained 17.7 % of the variation. It is important to notice that SQ1 colocalized with the QTL Fusarium4.

### Segregation of markers associated with QTLs

The study correlated the segregation of markers (closest to the QTLs) with the *Fusarium* resistance in the mapping population. To validate the effect of marker alleles on disease score in the offspring, a comparison was made for the two parental allele classes for each QTL derived from that parent. For this, infection area obtained from GFP test was chosen to represent the *Fusarium* resistance as it is the best quantified value, and also because the variance in the measurement is more likely to be normally distributed compared to the visual tests where high or low scoring individuals probably also have lower variance. Infection area was averaged for each genotype class and compared using the independent *t* test via PASW statistics 18. The phenotype means for the different alleles for the markers directly flanking the identified QTLs were found to be significantly different with *p* values from 0.001 to 0.022 (Table 3). As can be seen for QTL Fusarium3, SNP marker KN_12084C had the genotypes “A:C” and “C:C.” Mean infection area for individuals with genotype “A:C” (genotypes receiving the allele “A” from parent KN) was significantly lower than individuals that had the genotype “C:C” (receiving allele “C” from KN). In this case, receiving the chromosome from KN represented by allele “A” from the marker KN_12084C which results in offspring genotype “A:C” was correlated with a higher level of *Fusarium* resistance. Similar significant differences were also observed for all the other QTLs.

**Table 3:** QTL effects expressed as differences between marker genotypes for infection area from spot inoculation test with GFP

QTL	Marker	Offspring genotype	Mean ± SE	Sig.	Parental genotypes	
Fusarium1	KN_19786	A:G	37.56 ± 1.90	0.001	KN^a^	A:G
		A:A	26.16 ± 2.84		CA	A:A
Fusarium2	KN_23151	T:A	28.60 ± 2.83	0.022	KN^a^	T:A
		A:A	36.36 ± 1.94		CA	A:A
Fusarium3	KN_12084C	A:C	28.86 ± 1.96	0.004	KN^a^	A:C
		C:C	38.14 ± 2.49		CA	C:C
Fusarium4	KN_20195	A:G	39.53 ± 2.40	0.001	KN^a^	A:G
		G:G	28.61 ± 2.06		CA	G:G
Fusarium5	Ca_11976	C:G	28.59 ± 2.18	0.001	KN	C:C
		C:C	39.46 ± 2.20		CA^a^	C:G
Fusarium6	Ca_12006	G:C	37.86 ± 2.28	0.002	KN	G:G
		G:G	28.02 ± 2.16		CA^a^	G:C

Means of the offspring groups were compared using the independent *t* test option the PASW statistical package

^a^Parent segregating for QTL

## Discussion

### *Fusarium* resistance evaluation

The current study verified that *Fusarium* resistance in tulip is a quantitative trait. A clear continuous distribution of the infection has been observed in the disease tests. In other crops, both monogenic and polygenic resistances to *Fusarium* have been observed (de la Pena et al. 1999; Gervais et al. 2003; Matsumoto and Miyagi 2012; Netzer et al. 1977; Roman-Aviles and Kelly 2005; Sarfatti et al. 1989; Scott et al. 2004; Shahin and Spivey 1986; Spielmeyer et al. 1998). In the case of tulip, the genetic basis of *Fusarium* resistance was unknown.

In this study, the *Fusarium* resistance in tulip has been tested by soil infection using a mixture of three isolates and by spot inoculation using a single isolate (Tu67) with the GFP gene. Compared to other *Fusarium* tests in tulip described in the literature (van Eijk et al. 1978, 1979), the resistance tests applied in this study were much faster and needed less resources in equipment and bulbs, and although direct comparison on reliability is not possible, the identification of the QTL regions indicates that the current applied disease tests are a good and reliable way of testing for resistance. Because *Fusarium* can cause problems during bulb production cycles both in the growing season when bulbs are in the soil as well as during storage when harvested bulbs are kept in a cell at high temperature, the combination of the two tests mimics the two possible infection moments in commercial production. A mixture of three isolates was used for the soil infection since plants are always challenged by a mixture of isolates in natural conditions. From a breeder’s point of view, genotypes that are resistant to multiple isolates are more valuable. However, inoculation with a single isolate may be more straightforward to uncover the resistance mechanism. In the spot inoculation test, both single isolate and combinations of isolates were tested on parents and reference cultivars. The isolate combinations showed similar infection patterns as were seen with single isolates, indicating that a mixture of isolates could be used for screening the *Fusarium* resistance in tulip as well. A similar situation has been found for *Fusarium* resistance in lily (Löffler et al. 1995).

Soil infection tests were performed twice in 2011 and 2012. The correlation of the disease scores between the 2 years is moderate (*r* = 0.48), indicating a considerable environmental variation between years. The severity of infection in 2012 was higher than in 2011. The increase in infection severity of parent “Cantata” was significant (*P* = 0.003), while no significant difference between years was found in the more resistant parent “Kees Nelis.” The progenies of the mapping population also showed higher scores (grade 4–5) in 2012. This could be due to differences in the timing of the disease assessment and in environmental conditions. However, it is also possible that this is the effect of differences in the accumulation of ethylene that is produced by the *F. oxysporum*. Van Loon et al. (2006) reported that disease development was accelerated if plants were exposed to ethylene after infection. Different concentrations of ethylene cause variation in disease severity by influencing the disease development.

Infection area obtained from GFP signals may have an advantage over traditional visual scoring in performing QTL analysis. About twenty years ago, it was found that GFP expression can be used as a marker for gene expression and protein localization in living organisms (Chalfie et al. 1994). At present, GFP-tagged fungi have already been widely used to monitor the growth of these pathogens (Chen et al. 2003), study the infection pathway (Acquah et al. 2011), and the fungal–plant interactions (Buron-Moles et al. 2012; Maor et al. 1998; Valdivia et al. 1996). The approach provides an accurate monitoring of the fungus in vivo. The infection is quantified, and data analysis is easy to perform. Infection and progress of the disease are mainly influenced by temperature, humidity, and time of incubation. In the GFP test, eight progenies showed a large variation between the two replicate boxes. Four of these progenies were in the same box. Since the temperature and time of incubation were well controlled and uniform for all boxes, variation could be mainly due to humidity variation between boxes.

Using either a single isolate (spot inoculation) or a mixture of isolates (soil infection), a number of progenies showed lower scores than the resistant parent “Kees Nelis” or higher scores than the susceptible parent “Cantata.” It indicates a transgressive segregation with some resistant alleles also being contributed by parent “Cantata.”

### Genetic map

In the current paper, we have described the first genetic maps for tulip. The cross between *T. gesneriana* and *T. fosteriana* yielded mostly markers heterozygous in only one parent which segregated in a 1:1 ratio in the F_1_ progeny. A “two-way pseudo-testcross” strategy (Grattapaglia and Sederoff 1994) was applied for linkage analysis resulting in two separate parental maps. A total of 444 and 380 markers were analyzed for the female and male parent, respectively, of which 342 (77 %) and 300 (79 %) were successfully mapped. The KN map covered 1707 cM of the genome, and the CA map covered 1201 cM. Assuming equal recombination rates, this is similar to the result of a previous cytogenetic study (Marasek-Ciolakowska et al. 2012) which revealed that the total length of chromosomes representing the genome of *T. fosteriana* was slightly shorter than of *T. gesneriana*. For both maps, the number of obtained linkage groups (LGs) was more than the haploid chromosome number (*x* = 12). Similar results were found in the related crop lily (Abe et al. 2002; Shahin et al. 2011) as well as other crops (Alwala et al. 2008; Choi et al. 2010). Although higher numbers of LGs than chromosomes is a common finding in mapping studies, for tulip this may be enhanced by the species huge genome size of more than 30 Gb (Zonneveld 2009) and the possible presence of recombination hotspots as was suggested for lily (Shahin et al. 2011). Both tulip maps covered approximately 60 % of the estimated genome length. The proportion of unmapped markers in KN and CA map was 23.0 and 21.1 %, respectively. The presence of a considerable number of unmapped markers corresponds with a not completely saturated genetic map (He et al. 2014).

Since this is the first genetic map for tulip, we have used a stringent threshold (LOD > 5) for grouping markers to minimize incorrect assignment of markers and assure the quality of the map. This will have added to the number of ungrouped markers and the remaining of gaps. Both parental maps have a medium marker density (3.9 cM in KN map and 3.1 cM in CA map) in comparison with high-density maps such as tomato (1.2 cM, Tanksley et al. 1992) and rose (0.88 cM, Spiller et al. 2011), and low-density maps such as garlic (5.4 and 6.0 cM, Ipek et al. 2005), willow (7.8 and 8.0 cM, Hanley et al. 2002), and citrus (6.0 and 6.4 cM, Weber et al. 2003). Compared to the maps in lily, another monocot with a large genome (3.9 cM in LA map and 5.0 cM in AA map, Shahin et al. 2011) map density in tulip is slightly better.

The currently produced tulip parental maps provide an important basis to obtain a consensus map. In this study, however, parental maps have not been integrated due to the lack of sufficient bridge markers. A total of 52 bridge markers were available, of which 49 were AFLP markers that have a low information content being dominant markers. Therefore, most of the AFLP bridge markers (47) remained unmapped in at least one or both maps. In the end, only four bridge markers could be mapped in both maps. Obviously, this number is not enough for map integration. More markers, preferably SNP markers, are needed to join linkage groups belonging to the same chromosome, to saturate the genetic maps and to obtain an integrated map. One of the problems is that the parents may not have many markers in common, i.e., SNP markers polymorphic in both parents as the population results from an interspecific species cross. This may be solved by identifying different SNPs from the same common contig (assembled with high similarity, Shahin et al. 2012) and using these as bridge markers. An integrated map generated with a backbone of EST-SNP markers can be used to study synteny to lily in which *Fusarium* resistance is also mapped as a quantitative trait with a similar number of QTLs (Shahin et al. 2011). An integrated map is also an important basis for mapping of other disease resistance and ornamental traits.

### QTLs associated with *Fusarium* resistance

In this study, six putative QTLs associated with *Fusarium* resistance in tulip have been identified, indicating that tulip has a complex resistance mechanism against *F. oxysporum*. At present, very few studies have reported genes or QTLs associated with *Fusarium* resistance in ornamental plants except for a recent study describing six putative QTL positions in lily (Shahin et al. 2011). Of the identified six QTLs for *Fusarium* resistance in tulip, four were located in the maternal map and two in the paternal map. This indicates that not only the resistant parent (KN) contributed alleles to the resistance but also the more susceptible parent (CA). Because in tulip breeding and culture, plants will be in contact with *Fusarium* due to the wide spread occurrence of the pathogen, varieties must have a minimal level of *Fusarium* resistance to be successful. Therefore, transgressive segregation in crosses between varieties can be expected.

Correlation between markers and *Fusarium* resistance was first detected by Kruskal–Wallis testing and further validated by interval mapping and MQM. Not all putative QTLs from all disease tests were confirmed in interval mapping and MQM. This demonstrates that the *Fusarium*-resistance mechanism of tulip is quite complex with many genes involved, considerable environmental variation obscuring test results and the QTLs are consequently not very strong. In lily, Shahin et al. (2011) detected six putative QTLs by Kruskal–Wallis testing of which only one QTL could be confirmed by interval mapping. Also in that study, it proved necessary to perform disease tests in a number of consecutive years to obtain an accurate QTL mapping result for the strongest QTL. Three independent disease tests were performed in this study resulting in different QTLs that could be detected. Only Fusarium2, Fusarium3, and Fusarium6 were found in all tests, and they varied in significance for each test. Three QTLs (Fusarium1, Fusarium4, and Fusarium5) were only detected in the GFP test, although Fusarium1 was just below the significance level in interval mapping. More clearly, significant QTLs may be expected with a larger population size used. Remarkably, in the QTLs showing up in all three disease tests, the results of the spot inoculation test with the GFP-tagged *Fusarium* strain did not lead to significant QTLs in the IM mapping procedure, whereas with the GFP test QTLs could be found that were not detected using visual evaluation scores as phenotype data. Apparently, both types of disease tests are complementary and detect slightly different aspects of the resistance spectrum of the tulips. The combination of different disease tests has an advantage of detecting and confirming QTLs. As a physical barrier, bulb skin may be expected to have an effect on *Fusarium* infection success. Interestingly, a QTL for skin quality (SQ1) exactly colocalized with the Fusarium4 QTL, suggesting that a factor influencing skin quality also has an effect on *Fusarium* resistance.

In addition to the QTL analysis, direct association of the parental alleles segregating from the parent donating the QTL was checked. Phenotype means of the two offspring genotype classes for markers closest to the QTL were found significantly different and thus confirmed the presence of the QTL and shows which is the favorable allele segregating from the parent contributing to the QTL. The practical use of the obtained results for breeding relies on the distance between markers flanking the detected QTLs. Flanking markers of Fusarium4 (Ca_14945B, KN_20195) and Fusarium6 (Ca_13387C, Ca_12006) were at a relative small distance (5.6 and 5.9 cM, respectively). Markers flanking Fusarium1 (KN_5253, KN_19786), Fusarium2 (KN_36745 and KN_23151), Fusarium3 (KN_12084C, P31M54-26), and Fusarium5 (Ca_11976, Ca_15446) are at larger distances (8.1, 12.1, 10.4, and 9.1 cM, respectively). The genetic distance between the flanking markers should be as small as possible, so that the QTL would be useful in marker-assisted selection. Therefore, increase in map density and delimiting QTLs to shorter intervals is crucial to facilitate marker-assisted selection of *Fusarium*-resistant tulip genotypes.

## Electronic supplementary material

Supplementary material 1 (DOCX 685 kb)
