# Supplementary material for: Genetic mapping of resistance to Fusarium oxysporum f. sp. tulipae in tulip
Source: Mol Breed. 2015 May 7;35(5):122. doi: 10.1007/s11032-015-0316-3 (PMC4422938; doi:10.1007/s11032-015-0316-3)
Supplement: Supplementary file 1 — Supplementary material 1 (DOCX 685 kb) [file 11032_2015_316_MOESM1_ESM.docx]

Supplementary files:

Figure S1 Scoring of SNP markers genotyped using KASPar technology and visualized using SNPViewer2. A, expected segregation of <ABxAA> marker; B, expected segregation of <ABxAB> marker; C, segregation of SNP marker KN_24675, an example for presence of one null allele in one parent <ABxBØ>; D, segregation of SNP marker Ca_11914, an example for presence of two null alleles in one parent <ØØxAB>. Red and blue dots represent homozygous genotypes AA and BB, respectively. Green dots are heterozygous genotypes AB.


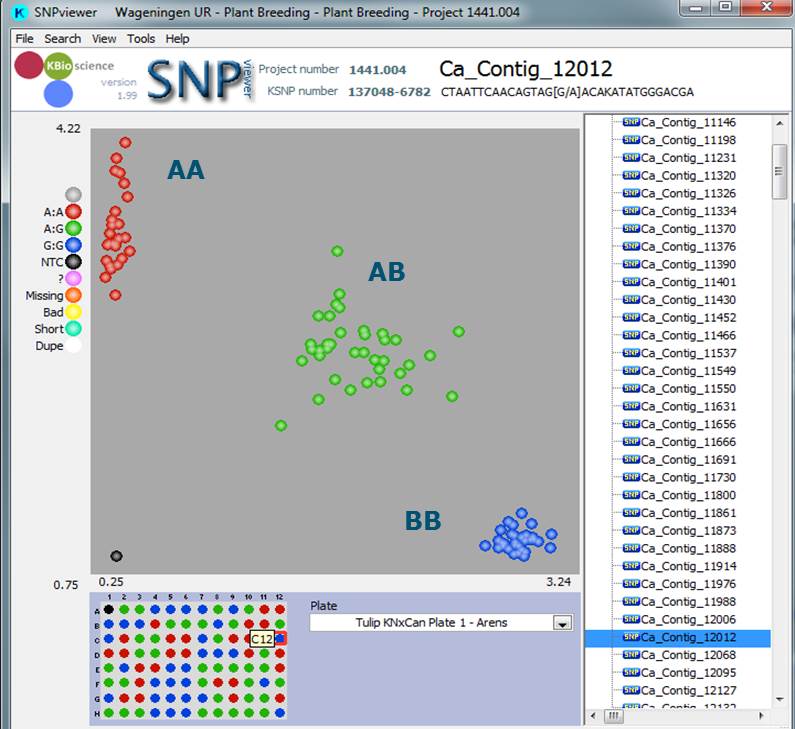

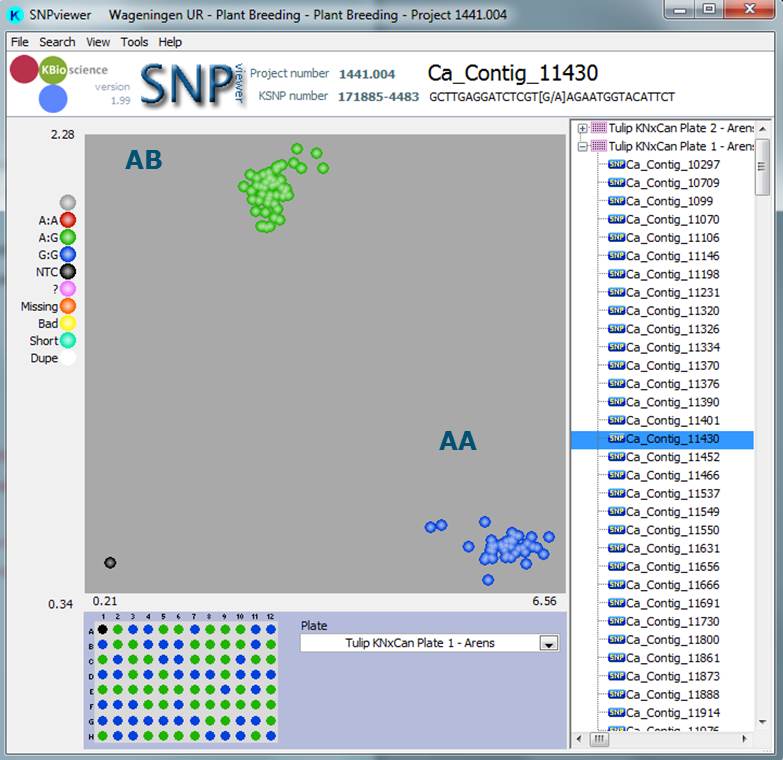


A

B


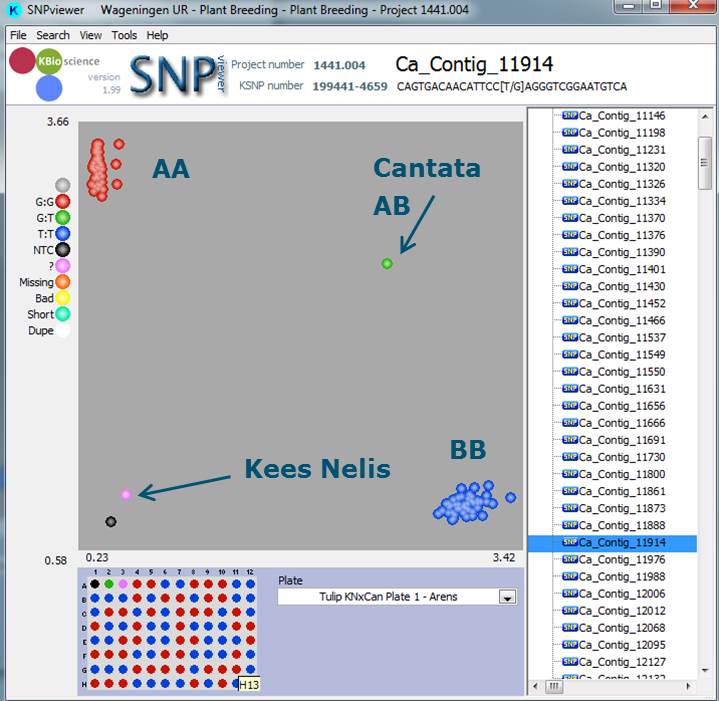

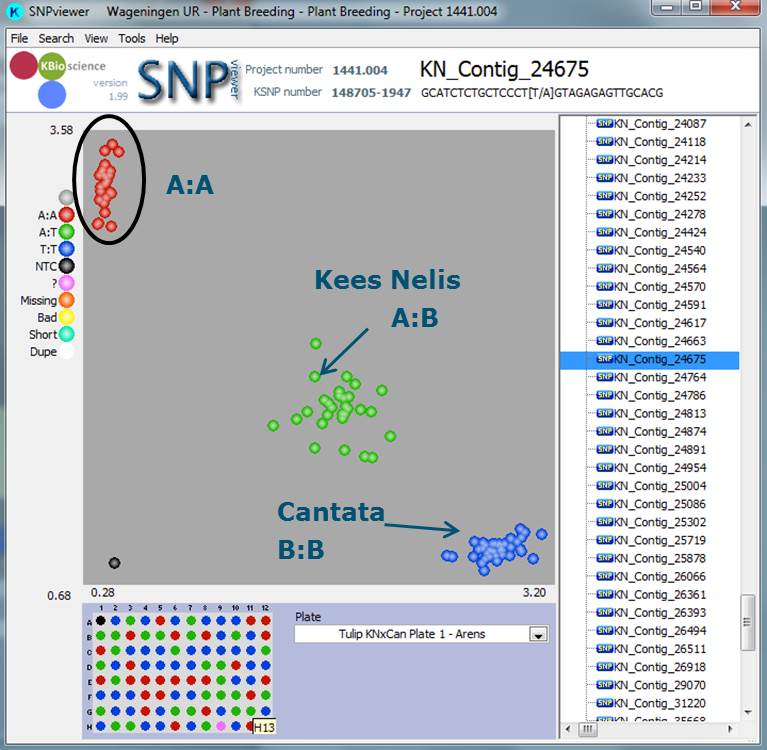


C

D

Table S1 Optimization scheme of PCR reaction condition for SSRs

| PCR parameters | Test range | | | | |  |
| --- | --- | --- | --- | --- | --- | --- |
| Primer (uM) | 1.0 | 1.5 | 2.0 | 2.5 |  |  |
| Mg^2+^ (mM) | 1.0 | 1.5 | 2.0 | 2.5 |  |  |
| Template (ng) | 2.5 | 5.0 | 7.5 | 10.0 |  |  |
| dNTP (mM) | 0.5 | 1.0 | 1.5 | 2.0 |  |  |
| Taq-polymerase (Dream) (U) | 0.15 | 0.30 | 0.45 | 0.60 |  |  |
| Annealing temperature (°C) | 52 | 54 | 56 | 58 | 60 | 62 |

Table S2 Scoring of SSR data based on segregation type

| Nr. segregating alleles | Allele | Genotypes observed in parents | | Genotypes observed in progenies | | | | Expected segregation ratio |
| --- | --- | --- | --- | --- | --- | --- | --- | --- |
|  |  |  |  |  |  |  |  |  |
|  |  | KN | CA | 1 | 2 | 3 | 4 |  |
| 1 | *l* | ─ | ─ | ─ | ─ |  |  | lm: ll= 1:1 |
|  | *m* | ─ |  | ─ |  |  |  |  |
|  |  | *lm* | *ll* | *lm* | *ll* |  |  |  |
| 1 | *n* | ─ | ─ | ─ | ─ |  |  | nn: np= 1:1 |
|  | *p* |  | ─ | ─ |  |  |  |  |
|  |  | *nn* | *np* | *np* | *nn* |  |  |  |
| 2 | *h* | ─ | ─ | ─ | ─ |  |  | hh: hk: kk= 1:2:1 |
|  | *k* | ─ | ─ |  | ─ | ─ |  |  |
|  |  | *hk* | *hk* | *hh* | *hk* | *kk* |  |  |
| 3 | *e* | ─ | ─ | ─ | ─ | ─ |  | ee: ef: eg: fg= 1:1:1:1 |
|  | *f* | ─ |  |  | ─ |  | ─ |  |
|  | *g* |  | ─ |  |  | ─ | ─ |  |
|  |  | *ef* | *eg* | *ee* | *ef* | *eg* | *fg* |  |
| 4 | *a* | ─ |  | ─ |  | ─ |  | ac: bc: ad: bd= 1:1:1:1 |
|  | *b* | ─ |  |  | ─ |  | ─ |  |
|  | *c* |  | ─ | ─ | ─ |  |  |  |
|  | *d* |  | ─ |  |  | ─ | ─ |  |
|  |  | *ab* | *cd* | *ac* | *bc* | *ad* | *bd* |  |

Table S3 SNP marker genotyping results in *Tulipa gesneriana* ‘Kees Nelis’ (KN_SNP) and *T. fosteriana* ‘Cantata’ (CA_SNP)

|  | KN_SNP | CA_SNP |
| --- | --- | --- |
| Genotyped SNP | 151 | 165 |
| Monomorphic | 17 | 24 |
| Polymorphic | 134 (88.7%) | 141 (85.5%) |
| Deleted SNP due to non-Mendelian segregation | 11 | 16 |
| Number of SNP used for mapping | 123 | 125 |
| Number of re-scored SNP used for mapping | 18 | 12 |
| Mapped re-scored marker | 16 | 10 |

Table S4: List of mapped SSR markers

| Marker name | ID | SSR* | Forward primer (5'-3') | Reverse primer (5'-3') | Product size (bp)* |
| --- | --- | --- | --- | --- | --- |
| SSRCA4 | Contig8508 | (GTT)10 | AGAATTTGTCTTGCGACAGT | TAGGGGTACCAATTTGTGTT | 325 |
| SSRCA4h | Contig7834 | (GAG)7 | CCTTTCTCAGTGAAGCTGAC | CAACACACAGACACACAATCT | 222 |
| SSRCA9h | Contig7271 | (CCG)7 | ACTCGTCTGTTGTTTCGAAG | TTACAGAGAGTCCTGGCATT | 180 |
| SSRKN10-2 | contig17568 | (CAG)9 | AGACCTCACACTATGGATGC | ATTGTAATGAGGTGCTGGTC | 192 |
| SSRKN11h | Contig2291 | (GAGAAG)4 | GAAGACGAAGATGATTCGAG | TGGGTTTCACTTAAACAGCT | 268 |

*Repeat number and product size from consensus sequence, annealing temperature 55°C

Table S5: List of mapped SNP markers (SNP indicated between brackets)

| Marker name | Marker sequence |
| --- | --- |
| Ca_Contig_10297 | ATATCTGAGGGTACTCGAGTGTTAGTAAATGTTTGGGCGATGGGAAGAGA[C/T]GGAGATGTCTGGAAGGACCCCGAGCAATTTATGCCCGAAAGGTTCTTAGA |
| Ca_Contig_10709 | ACGGATGATGCACATATACGAGGAGAGTCGGATCATATTAATCTGATTAA[T/C]GGGATGCCTGTTGGAAGGGAGCTACTAATTTTGCATAGCTCTGTCTTTGT |
| Ca_Contig_11146 | AGTTTTGTTGCCCCGGATAATTCTCAGTATTACTATCAGCCGCAGCATCC[G/A]CAGCAGCCAGAAAGCATTTCACGGAGTCCCTATGGTGATGGTTATCAACA |
| Ca_Contig_11198 | TGTCGACATCCTACATTTGCGACTTACTTCCAGGAGCCTTAACCTCCAGC[A/G]ATTCGAGAGATTATTGCGTGGGAATAATGCTAAGAAGGGTATGCGGATTT |
| Ca_Contig_11231 | TTGTGGGCATCAACTAGCGTTAAAAATCCTGCATATCCWGACACGCTTTA[C/T]GTGTCTCCTCTCATTGGACCTGATACGGTCTCAACAATGCCTGATCAGGC |
| Ca_Contig_11401 | TCAGATCCTTCAACGGTTCTCTATGGACGACTCAGCATGGGCATACATTA[T/C]TCGAGAAGCCCGCAGTGACACACGTATCGAAAGCTGCTTTACAATCAGTT |
| Ca_Contig_11430 | ACAAGGACCGGCACAGACATCACCCGTACTCGTGAGCTTGAGGATCTCGT[G/A]AGAATGGTACATTCTTTCAAGTTATATACCAAGTCCTTCTCTGATCCCAA |
| Ca_Contig_11466 | CCACCTAATGGGGCTATTGGTACAAGAATATTGGAGTTTCTTTTTCACTC[G/A]AGATCCGATTTGGTAAATATTAGAAGTGTTGATGAAGCAGTTGTGATTGG |
| Ca_Contig_11537 | GACATGTTTCACAACTCTCAATGGGCGACCCAAGAAGGCCTTGGTCTGAG[C/T]GTAAGTAGGATGCTCTTGAAGCTGATGAATGGGGAAGTCCAGTATATCAG |
| Ca_Contig_11691 | CTTTTTGAACCTGTCCGCAAGACCCAAGATGCCATGTTCTTCATAAAGAC[A/G]TCGAATGATATGTGGATGCCGTGCGGACCAAAACAGCCTGGAGCTGTCCA |
| Ca_Contig_11730 | GTTAAGCAGCACAAGTATAGTAGGAAGCGGCCAGATGAAGGGTGGAATAT[T/C]ACAGTTGAGAAGCTGGGCCCTGGTGGGAAACGCGGAGGGGGAGGCGGCTG |
| Ca_Contig_11800 | AAGGACTGTGAAATGTCTGCACCCTGTGGGACGACAGTTCTTTATCCTAC[C/T]GGAGGTGGCAATATGCACTGTTTCAAGGCCATAACTCCTTGCGCTCTCTT |
| Ca_Contig_11914 | GACCAATTATTCAGCAAAGAGATAAATCAAGCCTGCAGTGACAACATTCC[T/G]AGGGTCGGAATGTCATCTGAGGTSGCAGATCCATCTTACCTGCAGTTTAA |
| Ca_Contig_11976 | ACCCTAAGGTTCTGGAATGTCTTCCCGTCTCTTAAATCGCAGAGTACCGG[G/C]AGCGATATAGGAGCATCGTCTCTAGGTAGATCCTACATCCGGTGAGGTCT |
| Ca_Contig_11988 | ATTCAAGAGTTTATAGATGCATTAAGCCAAGCTGGAGATAAGCTWGTTAT[A/T]GCTGAATTTTACGGCACTTGGTGCGGTTCCTGTCGAGCCCTTTTCCCAAA |
| Ca_Contig_12006 | GGGTCGTCGCCGAGGGCTTCGTTGTCGGATAATGGGAACGTGGGTGGGTC[C/G]GAGTTTGTCAATGCATCGAGAGCATTTTCTGAGCCTCAGGACAAGACTTC |
| Ca_Contig_12068 | AGGCGGCCTACGATAACTGCAACACGGCTTCATACATCCAGAAATACGAC[A/G]ATGGGAACACCGTCGTTAAGCTCGACCGATCAGGCCCCTTCTTCTTCATC |
| Ca_Contig_12095 | GAAGTTGAGTTGGAAGGAAGCGATTCGAGGCACACTCTTCTGGAATCTTC[G/C]CTCCGTGAAGTCATGTTTCAAATCATAAAGTTTGTCAATGACAAGAAAGA |
| Ca_Contig_12132 | TGCCTATGGACAAAGTGTATACTCCCGGTAAACCTTGAAGGAATAGTGTT[G/A]AGATGTGAAGACACATCGAAGATCGATGTTATTCCGGTCTAGTGAGGTGC |
| Ca_Contig_12149 | TTTGGAGAGTGTTAAAAATTGATAGAACGGAGGCTTCCGAGCTATTCATC[C/A]GCGAGGACTCCACCGCGTACTCAGAAAGTGAATGCTATAACCTKTTGATG |
| Ca_Contig_12393 | CTCCACCATTACACTGACTGGCTGCCGGATAACCTCAACAAGGGGAACCT[A/T]GAGTACTTCTACTGGTTGGTAACTCTACTCCAGATAGTGAATCTTGCGTA |
| Ca_Contig_12438 | GTCATAATTCGTGATGCAGCGGCTCTTATAGAAACTGCAAGATCCTTAGC[A/G]TCACAACTAAAATGGGATGCACGCATGATGGATCTYGAGAGTTACAGCAA |
| Ca_Contig_12474 | GTCATATACGTCACCTAAGAATCCAAAATCCAGGAAGTCAGTTCATTCTG[C/G]AGAGCAATCACCATTGCGTGCAAATACAGAATCTAATGGAAGCGGGTACC |
| Ca_Contig_12486 | CACTCTGGATCTCATAAGAAGATGCTCATTTTCAAATTTGCACTTCCAGA[C/T]GCCAAAAATATGGCTGACATGACGAGATTGGTTACTCTTGTACCTTATTA |
| Ca_Contig_12523 | TATATGTCCTTCAAAAGGTCTCTTCTTGTACCGAGGCAGCTGTATGAACA[C/G]AGATGTGTTTAGCCATAGTTATCTGCGTGTATCACAATGAAGCAATATCA |
| Ca_Contig_12552 | GTCGCTATGCCTGCTATGAATAATATTCTCTCGAAATGGATCCCAGTATC[G/A]GAGAGAAGTAGATCTTTAGCACTTGTGTATAGTGGGATGTACCTTGGCTC |
| Ca_Contig_12573 | TTTCCTGAAAGAAGCCGTGAGGGCAATAAATTCACTGTGGCTGAGAGACC[T/C]GTGAAGCAGGCGAGAAGAAACTACTGTTCTGATTCCACTGAGTCAGTGAT |
| Ca_Contig_12635 | TACAGAATTGATTTGAGCATGGAATGTTACGAGGGTTTGCTCGGGTGGAC[T/C]GGATCTTGAAGAGTTAGGGGTTTAGATCATTGATCGGAGCTGTGAAGTAC |
| Ca_Contig_1267 | TCTGATGACTCCGCGCAATCTGAAATGGCACTATCCCCGTATGAGGAACC[G/A]CCATTGTCCATACTACTGCCAATTGTCGTAGTACCCGCTGACACAAACCG |
| Ca_Contig_12690 | AAAGTTCTAAGATGTTACTTATTGAGGGAAGGCAGAAAGGTCTCTCCAGG[T/C]TAGATAGGAATGATACCAAACCCCTTACAAAGTCTGAAGTTGATGCTGAA |
| Ca_Contig_12702 | GAAGTAGAAAAAGCAACCAAGAAAGTCGACAGTGAATCATCGACCGCTAT[C/T]AGGGGTAATGCACTGCTACGATACAAAGAGAAAAGGAAAACTCGCAGATA |
| Ca_Contig_12757 | GCCTCAGGTGCTCATAGGAAGGTTGGTGCGGTCCAGTTCGGCATGTCACG[T/G]ATTTATTGAGGAGTTGAAGGACCAATCAGCGGTTGWATCCACTGCTGTTC |
| Ca_Contig_12772 | AACAGTACTTCCCCTGTCCATTACTCCCACAACTGGAAATTTTGCCAATA[T/C]GGGTGCACAGAGAACTATTTCACTGCGCACTACCAGTCTCCCCGGCAAAT |
| Ca_Contig_12792 | GAGATGAGCTGCACAGGATGYTAAATGAGGATGAGCTACGTGAAGCCGTG[C/T]TGCTTGTGTTCGCTAACAAACAAGACTTGCCGAATGCAATGAATGCTGCT |
| Ca_Contig_12810 | TTGTTTCGATAACGCCTACCTTGATAAGCAAAACCCAAGCTCCAGAACCA[G/A]CTCTTGTCAAAGACGACTGATTGTTGTCGTGTCTGTAAATATCAGCTTCA |
| Ca_Contig_12899 | TCAATGCATTTGACAAAGCTGCAGACCTTGATGTTTTGAATCATCCGGTT[C/T]TGAACTTCTTATACTTCCTGCTCGTGGAAATACTCCCTTCAGCACTGGTC |
| Ca_Contig_12914 | TACCTGCCGATCACAGAAGTGATTAAATCTCCCAGGTGCGTCGACCCCAA[T/C]AGCAGAATGTGGCACCGATGCTTGACATCCAACGGCCAGCCTGACTTCTG |
| Ca_Contig_13039 | TGATAGGAAGTGAAGATGTTCCATCACTCGGGGAATCACATCAGGTAATC[A/T]CAGATCAAGGTGAACTCGCAGAAGAAGTCATCAATGGCAGGATGGATTGG |
| Ca_Contig_13055 | TTCTGCCCGGAAGGGCCTGAGCAAGTTAAGCGCCTGGAGGATGCCTGTCA[T/C]ACTGAAGGTTTCATCGTACAGACGGCCACCGTCGTTCCTCCTCTCCTTAC |
| Ca_Contig_13301 | ATCTTCCCGGCTACGCTCGAGACTATCACGAACATCGGCAAGGTTGTGGA[T/C]AGCCTGTATGTTAGATCTGCCAAGATCATGGCTTAGGACGAGGCTGTATG |
| Ca_Contig_13341 | GGCTTRGACATATTGCAGCCTGACAATAAGTGGAAGCATGCGTATATTGC[G/C]ATCATCGGGACGCTGGCAGGGATTGCATTGTTTCTAGAAGTGACTACATG |
| Ca_Contig_13360 | GCAACCGGGTTCTCGCAAACATTTCAGGAGGCTCTATTGATGGATCCCAA[G/A]CCATTCCAGAATGTCACACAGAGCCCCAGTACTAGTGTTCCGGCACTCCC |
| Ca_Contig_13387 | ATTTAAGCATAACAAATAAGATACAAGATCTTCAGGAACTAAATGGTCTA[T/C]CTGAAGTGAAATCGGCCCATTCATCCGTACACAACACAGCTGGAAATGGA |
| Ca_Contig_13392 | TATACGTATTTGGAGTCATCAAAGGTGATTTACCAGGAGCAGGGGAAGTT[A/G]AGTTTATGCAAGTTTGGTCCATTTTTCGTTGGCAGATACCAGGTCTCTAT |
| Ca_Contig_13399 | ACCTGATTACCTCTCTTGTGCAGCACCTGTTTATAATCAGATGCAGTTTG[C/T]CGTGTATATAACATTCATTTATTCAGCTGTGAGTATAGGTCTTTTTTAGG |
| Ca_Contig_13422 | CAAAGCAGAAAGATTGTCCAGAAACTGGAAGGCCATACTGACACAGTCAT[T/A]GCAGTTTCTTGTCATCCGACGAAGAATATGATTGCATCTGCTGGACTGGA |
| Ca_Contig_13475 | GCAGAGTCTCACAGATGGCCGACTGTTGGCAAATATAAAGTGGATGTCAC[A/T]TCATTCGAATCACTGGCATTGCCTGAATTGCAGGTTAAAGAGGATATAGA |
| Ca_Contig_13521 | TCATTAGCTCACTATTTTTGGATCCAGTGTAACCGTTCATAGTTCGAGGG[T/C]GTCAAAGGGTACAGGAAATTTTGCATCAAATGAAGCTTTGCAAGAATCAC |
| Ca_Contig_13566 | AGGTTAAAGCAAGATAAGGAGAATCTGGAGCAACAGGTGAAGATGTTGAG[T/C]GTACGCCCGAGCTATATGCCTCACCCACCAGTGTTACCAGCTGCAGCGTT |
| Ca_Contig_13679 | GGAGACTCATTACTTGAGTGTGCATCTATTGCTGATTCATGCAAAAAGAT[T/A]GTGGCGGTAGGGATCAACTGCACTCCTCCCAGATTCATTCATGGATTAAT |
| Ca_Contig_13689 | GAGAGTYCAAGTTTGGGAGGATCATGTCATGGCGAATCGAGGACCAATGT[T/G]GTAGACAGAACCATAGCAAACCTCATGTTTAACAATCCATATTTTCAAGC |
| Ca_Contig_14608 | GCGATGAAGAGGATTTCAAAGATTTCTGTTTGCGAGCCTCCAAGCTGGAA[G/C]ACTTGTCAAACGGGGCACCGTTGTTTACAGTAACTCAGTCTCTTCGGCTG |
| Ca_Contig_14725 | AGAGGTCATGTGAGGAACCTTAGGCGCATGGTCGCCCAGGAGATGATGAT[T/C]CAGCAAACGCTACAGGAGATCCAAGGGATGATTTCACCKTTGCGAGTAAT |
| Ca_Contig_14822 | CCTACTAAGTACGCMGCAATGTTAGCGGAGAAAATGCAGAAGTATGGTGC[T/C]ACAGGATGGCTCGTAAATACCGGATGGTCTGGTGGAAGGTATGGTGTTGG |
| Ca_Contig_14945 | AACAAGAATGTGTGAGACGACGTGCCTTGAAATCAGTCGAGAAAAATCGC[T/C]ATTGTTCAGGAGCTGCTGCAAAGGATGCCATTGAAGCTGTCTGGGATGTC |
| Ca_Contig_15101 | GCAGCGGAACTAGTCAAGCATCTCTTGGTTGTGCCGGACAGTCCTTCAGC[G/A]CGCGTAATGGATCTTCCAACTACAAGAAAGATAGCATTGAAGAACAAGAT |
| Ca_Contig_15185 | AGGGTGTACAATCTTGGGAATACGTCGCCGGTGCCGGTTTCGGAGTTGGT[G/T]AGTATTTTGGAGAGTCATTTGAAGGTGAAGGCGAAGAGGAAGGTTGTGAT |
| Ca_Contig_15362 | GGCACCACATGTATAATTTTCGGTCAAGGCCACCAAATCATTTCCAATAG[T/A]CCCCTGAGGGTTAAGCTTCCCGTCCTTAATGCCACAGAAGAACATCTCAA |
| Ca_Contig_15446 | ATGCAGTTGAAGGACACCGTAGCGCTCAAGTAYGCGGAGCTAGTGTACGC[T/A]GGCAGGTGGTTTGACCCCCTACGTGAGGCCATGGACAAGTTTATGGAGAA |
| Ca_Contig_15634 | CAAAATCAAATGGAGTGGTGGAAATGTTTGATAAAAGGAGACCCAGAAGT[T/C]GACACACAGAAAGTGGAACCTGAGAATAGCAAGCTGTCTGATTTGGATCC |
| Ca_Contig_15858 | GAAAGTGGGGGGATCATCAACATGATGGGAACATATCAAATTGCATTGGT[A/T]GCTCATAGCATGAACACGCCTGTCTATGTGGCTGCTGAAAGTTACAAGTT |
| Ca_Contig_16280 | ATGCTCAACAAAGCACCGGCATTATCAAATTCTCCGCAATAGTTTGGAGC[T/G]GTAAATATCGTAACTAATCTCCTTTGAGCAAAAAACTCGTATCCATCTTC |
| Ca_Contig_16557 | TCAGCACGAAAAGCTTTTAGAAATCAGTTGGCGGGATCTTCAAGTGCAGC[C/T]GTAGAAAAGTCATTGATGTGTGGATAGACCGCCTTCTCATCAAGCTGATT |
| Ca_Contig_16619 | AGCCTTTCACTCCAAAAAGTAATGTCCCAGTGGGTTAATTCATTAGCCTC[C/T]RAAGCACCACTTTCCCCAGCAAATGTCTTCAGAGATTCTTGGTCTTGAAC |
| Ca_Contig_16623 | GCGGTATGACCCAATTYCCAGATACTCGGTTATAGCATTCATAACATCAG[T/C]GTGCCTATCAGATTCTTGCCTAATGTCAAGCCTTACAAGAGACAGTCCAA |
| Ca_Contig_16630 | GAGGTACCCAGAGTTTGAGTAATCCCACCAATGAAACTGAAGCATATCCA[G/A]AGAAGCAACATCCATCCTCTTCCTTGATATGTCGATGTTCTCCTGAACAT |
| Ca_Contig_16660 | TCGAACCTGGCGCGAGTGAAGGTGGAGTAGAAATCAATGCCCTCATAGAG[G/A]GAGTCRATCTCGATTGTAGTTTGAGCAGTGGAAGAAAGGGTTCTCTTCGC |
| Ca_Contig_16694 | GCTGCCATGTTGCTTGTCACATCAAGAATTATCTTGCCTTTGCTTCCGTC[G/A]TATCGTCGTTCCCAAATTTGCAACTTGAGATCACCATAGCATGTATCTTT |
| Ca_Contig_16795 | GATGTTTGGAACTTGGCCGGTGGCTATCTTAAGTCGCTGCACTTCTTCTC[T/G]CAGAGCTTCATTTAAGGCATCACGAAGATGAGCCTGTTGCTCCATCGCTT |
| Ca_Contig_16891 | TACATGGGCTTTCTCTTCGACGACTCGCCATCGGCACCAGCAATCACACC[G/A]TCATTCTGGGCAAGGAACTGCTTCCTCATTGCCTCCAACCTCTTCTGCTC |
| Ca_Contig_16920 | TTCTTCTTTGATTTCTCTCCATGAGATTTCTCAGACCCTAACGAAGTATC[G/A]GTAACCTGACTTTCACGTCCAGAAGCGTGAGATTGTTCATTGGACTTCAA |
| Ca_Contig_16926 | TGAAAGGGCAATCCAGCTCCWGATCACGAATTAAACAATCTTCTCTGTAC[T/C]YTGAATTCGAATATTTTGGATGTTGCTGAAAGGAAACACTTTTCCACCAT |
| Ca_Contig_17010 | AACCTACAGTTTCTTCCTAAAATTTCCTCCCGTGAATACTCCGTCAGTTC[G/A]AGAAAGCTGTCTGATGCATATATTATTGGATTATCGGGTAGTCTAGGATC |
| Ca_Contig_17031 | TTGTCATGGGCCCAAAAGCCRCGAACGTCTATGAGCATACTCGCAACCGT[G/A]CCTCCTTGCTTGCAGGATAGAAGATCCGAAAGGGATATCCTAAGATGGTC |
| Ca_Contig_2212 | AGACGAGGGTAGAGGAGAGGGATAAAGTGATAGAGTCGCTGGGGGAGGTT[T/C]TGGAGAAGGCGGAGAAGCTTGAGACCGCGAAACCGGTTAAAGTTGGCGGT |
| Ca_Contig_2419 | GTCTTCCCAATGTCGTTCTTGATGGAACAAGCAGGAGGTCAATCTTTTAC[C/G]GGCAAAGAACGGGCCCTMGACTTGATTCCTACCAAGATTCATGAAAGAGC |
| Ca_Contig_2533 | TGCTGCTTCAGCTACTTAGGGAATATACGGTGAAAAATTCAATTAAATAT[T/C]AGAGTTCTTGATGAASAGTTGTCCGTCACCGAATACTCGAAGAATCAATT |
| Ca_Contig_2582 | GGGATGAAAAGGACACGTCTGACGAGCTTCATGAAGAAACGTCAGCAGAT[T/C]CTATTTCCAATGAACCAGTGAGCAACGAGGAGGAASCCACAAGTAGCTCA |
| Ca_Contig_2583 | CAAGCAGCTATTGAGAAGCAAGGTTTGAACCTGTTGGCTTTGGTGAATAC[C/A]GGAATCCAGTGCAGCCTCAGACAGCTATTAGAATATGGATATTTCCATGC |
| Ca_Contig_2678 | GACAACAGTGGTACCATCGACTACGGGGAATTTATTGCTGCAACAGTACA[T/C]CTCAACAAGTTGGAACGCGAAGAACACTTGGTGGCAGCATTTGCTTATTT |
| Ca_Contig_2685 | TCTATGAAATGGTAGCAGTCTTGAGCGTGTTAAGAGAATATCTGTGTTGG[A/T]GCACGATACGTTATTCTGGCCTGAGAGACCCATCATGCTGAYCAAGAACT |
| Ca_Contig_2736 | GTATGGCTCCAAAAGGTTTATCAAGCTTCTTGCATGGCTGATAGCTATTA[T/C]CCAATCTCTCCTTATAATAGCTTCTCGAAAGCATTACACAGTAGATGTTG |
| Ca_Contig_3184 | GGTATTCAGAACCCAGCTCAGCCGCGAGCGTGTTCAGCAGGGAAGCAGCG[G/A]CAGCAACAAAGGAATAGATGTGACATTGAACTTCAGACTGTGACTCAAYT |
| Ca_Contig_3298 | AGGAGATGATCCATTACGAGAAATCGATCTACAAGGCTCAGAGAAATGTT[G/C]TACTTTGCTTCGGGACATGTTTTCTTTATTGGTACATCTACCGTGCTTGC |
| Ca_Contig_3629 | GGGAGGATATGYATAGAAGGTGAAAGAGTGGCTCTTCTTGGATATGGATC[A/T]GCAGTTCAAAACTGTTTGGCTGCAGCTTCCATTGTGTCACAGCACGGCTT |
| Ca_Contig_3957 | CAGCCGAGGACGACGCTGTCGTCGTTGTTAGGGGAGCTTTCTGGGTATAC[A/G]CAGTTTACYATTGAGCAGATGCCGATGTCGGGGAAGATCTGCCTTGCTAG |
| Ca_Contig_3968 | AAGAAGAAAAAGCCCAAGAGGAAGAAAGATCCTAATGCACCAAAAAGGGC[C/A]TTGTCTGGTTTCATGTTTTTCTCAAATTCTGAAAGAGAAAATTTGAAGAA |
| Ca_Contig_4218 | AAAGCGGTTGCAAAGTCAGCTGTTCTAATGAATCTTGAATTTAGAGGAAC[T/C]GCCTCTGAAGATATTGGAAGGCAAATCTTGACATATGGGGAGAGGAAACC |
| Ca_Contig_4427 | GGGTGTGCTTTTACATGATTTTTGTTGTGAGACTTGATTCAAGAACTGTG[G/A]AGCCGCACCAGTTAATTTCCATGCAAGTAAGCTTYATCTGATGAACAGAA |
| Ca_Contig_4464 | TCCGAGATGAAGTTTATAGGGCAACATGATGATGCTACCAAAGACCGCAC[T/A]GGGAAGTATATTTTGATAGGAGATSAAGAAGACTCGCGATTTGGCATTTA |
| Ca_Contig_4658 | AACATGAGAGTACTGTTGAAGCTCTGATTGATTAAGCCAGTATAGTGATG[G/A]AGGATGTTTTTCCTGTGTTCACCTGATGTTTTATCGAAAGTGGAWTTGAC |
| Ca_Contig_4752 | AGGGCAAGCAAAACCATGACAGAACAGGAAGCTAGGCAGATTTTAGGGAT[T/C]AGTGAACAGGCACCATGGGAGGAAGTCCTGAGGAAATATGATACCTTGTT |
| Ca_Contig_4859 | CTGGGCAGAGGTTTACTACCATTAATGAAGAGTTTGTAGCTCAGGTGGAG[A/G]AGGTGGTTTCGGACAAGGAGGCAAAGGTTTTGGTGGCATGCGGAGAGGGG |
| Ca_Contig_4888 | AGTCGTCCAGCGCACTACCACGTCTTRTGGGATGAAAACAAGTTCACTGC[T/C]GACTCGCTACAATCTCTCACTAACAACCTATGCTACACWTATGCGAGGTG |
| Ca_Contig_5615 | TCTTTATGCAGTGTCTATTGGATCTTTTGTTTAAGTTGGTTCGGAACAAT[G/A]TTTGTTTATGTTATGGTAACCAAGTTTTGTGTTTACTTATGCAAGGATAA |
| Ca_Contig_7026 | TGGGTGAGGCGTTCATTGTAGATAAGTATAACAGACCCATGCCAAAGCTA[C/A]GGAGCTCCAAAGCAGAGACTGCAGATAGCAAACAAGATTCAGTGCCTCCA |
| Ca_Contig_8355 | AGTCCACACACAACATCCAGGTTAGGAAGGCCCTTTCAGGTTAATACAGA[T/G]ATATGTTGGTGCCTCGTCCCAGTCGCRTTTTTATCACCAAGATCCTTGCC |
| Ca_Contig_8430 | AGCTTAGGCATGGATGGAAGACTTTTCATCATCGGATTTCAAGGAGGCAC[C/A]GTTACAGAAGTGAATCTTTCATGCCTGCTTGCAAGACGCCTTACYATACA |
| KN_Contig_10239 | AATTTGATATTCAAGACTGAAGCATTCCTCAGCTATGTTGTACTCTCTTC[T/C]TGGAGGGACTCCATTGTGGTGCTCAAGAGCTGCGAGGGTCTTAGTCCATG |
| KN_Contig_10436 | CGCGTTGAGAGCAGGGATAAGTATAGTAGCGGACGCCTTGATCGATATGA[C/T]CGTTATCCGCCTGCTGGTGATAAGTATGGTGCATCTGACCGTTACCCTCA |
| KN_Contig_10730 | AAACCGACCACCGAAGTTGGCTGGAGCTTTCTGTGGGTTATCTTCCTTGG[T/A]CTTGCTGTTGCTGGAGTGGGAGGCTATGCAGTCTACAAGTACAGAATCAG |
| KN_Contig_10934 | CCGTGTAAAGATTCCCATGTTTCTTCTAAGCACCATGTTTCTTCTAAGCA[C/T]ACCTTGTCAGATTCGAAGGAACATTCTTGTTTCACCACCTCATAAAGCTT |
| KN_Contig_11086 | AAGCTGAAGGTGGCGGCGTTCTGGCAGGACTCTAGGCCGACATCGATCRA[T/G]ATGGAGCCCATTGCCGGAGTGGAGGAGCTTGAACAGATACTCARCAAGGC |
| KN_Contig_11108 | CCACTTCATCCCTTTCAACAGGCTTCTCTATTGCGATCACCTGTTTCACC[G/A]CAGGCTCAAGCTCGCCCTTCTCTATTGCGATCACCTGTGTCACCGCAGGC |
| KN_Contig_1129 | GGCCCAAACCAGTTTTCATTCCAAAACAATAAGAACGACAGAGGCAATCA[T/C]GAGGCTTGCTTATGGTTGGGTGCTCATGGGACTGAGGGTTCAAAATACTT |
| KN_Contig_11303 | GGCCGATTCTTGAAGGTCTCAGAAGCTTCTGCCAGCAGAAGTCGTAGCAC[G/A]ATAATCGTTCCTGCGGGGAGTTCTGGTGTGGAAGGATGGACAGCATTCAG |
| KN_Contig_11637 | AGGATAACACCTTTGAATTTATTGTCAAGTCACCATCTGTTACCTGGTAC[T/C]TAAAGAAGGCTGCCGGGCTTGAATTCGGCAGCGGCCGCCCCGGCCATGCG |
| KN_Contig_11731 | AGATTTTGGACCTTTGCAACTGTAAAGAAGATGTTAGGAAGAGGGTTCAC[G/A]CCGTGATCGATAAATTTGCCGAACGCGGCCTTCGCTCACTTGGTGTTGCA |
| KN_Contig_11900 | GACTACCTCGACGAGTCAATCCTTTGGTCGGAAAGCAATGACACTGGTGC[C/T]GGCTTTCGATGCATCAGAATGGCGAACAACATCCGTCTCAACTTTGATGC |
| KN_Contig_12084 | TCCATGTAAGGTGGAAGAAGATAGTAGCGGAAAAGCTGATGTTAATGCAT[C/A]TGCAGTGGAGACGGATTCCTCTGATACAGAATCCTCAGTTGAAGAAGACG |
| KN_Contig_12168 | TTCAGAGCTGAACAGTCTTTAGATTACTATAACGAGGAAATGTTTCACTG[T/C]AGCGAGGGGTCGCCGGTTACCAGCAGCTTAAATGCTCTAGAAGATCAGTC |
| KN_Contig_12229 | ATTAGTACGCCGTTGTACCAGACATCCACGTTCGAGCAGCCTTCAGCAAC[A/G]GAAATGGGTCCCTATGACTACACCAGAAGTGGTAATCCTACTCGAGATGT |
| KN_Contig_12299 | GTTGCTTTAGCAACGTCATTAGGGGAAGTTGAATATGATCCATCAGTCAT[T/C]AGCAAAGATGAAATTGTGCTCGCAATTGAAGATGCTGGATTTGATGCTGC |
| KN_Contig_1232 | ATGTATACCAGTCAAAGGAGTGAGAGCATGGTGGCGTTCTTTGATGGGTA[T/C]GTTAATTCTAAAACTACCTTACACCAATTTGTTGAACAATATGGAAATGC |
| KN_Contig_12356 | ACACTGGAAGGTAGCATCACAAGTGGAGGGGATGATAAGAGAGGTGAGGA[T/C]CTGTTCGAGGGCTCGAGTGGATACATGGAGTGGCTTGATTCCAAGGATGC |
| KN_Contig_12552 | AGRGAAATTGAGAAGGCACAACATGAGGAGGAAATGGCAATGCTAGCCAG[G/A]GAACGAGCAAAGGCAGAGTTCCAGGACTGGGAGAAGAAAGAAGAAGAGTT |
| KN_Contig_1280 | GGTAAGTTTGGGYTGCTGTGTGGTACTGACGGGCTTCCGCATTTGATCGT[C/A]AGCGGGGATCAGCGGCACTGGGGGGGAGTTCATTACTCCTGGGGTGCTGT |
| KN_Contig_13138 | AATATCTTGCTGTACAAAGATGATGGAGAACTTAAATTAAATTATGCTAG[T/C]TTGTGGATAATTGCCCGCTTTTGTTGATAGAGGTGCTAATAGCTGACTTG |
| KN_Contig_13398 | CATGGGAGGAGCATTGATTGGGGAGGTGAAGTAGACGGGAATTGCTACAA[T/C]CAGACTAAGCCAATCGAGGACCTGAGCTACTGGGGACCGAGCACTAGTAA |
| KN_Contig_13923 | GTAACTGTCGGTGCATCATTCTTGTCACAAACTATAGCCCTGCAAGACTC[C/A]ACGACAGTTAAGTTTGAAATATGGGATACTGCTGGGCAGGAGAGGTATGC |
| KN_Contig_13983 | GATTTCATTGGTGATAGTGAAGTAGCTTCTAAGGTTATAAGTGAAAGTAA[T/C]AACGAGGGGGAGGAAGCAAGAAAGTTCTTGGAAGATGTCCGTGTCACATT |
| KN_Contig_1404 | TGATGCCGAGACTCAGAATAAGATTAACACACTGAAGCAGGAGGTCAAGG[T/A]GAGCCTTGCAGAGGCTATGAGCTCACCATCATTGATTGGGAAATATGAGA |
| KN_Contig_14298 | AATCCCAGGATCCATGATGCCTTCACGGGCATGGGAGATTGCAGGAACCT[A/G]GTGATCCCAGGCGGGAAATTCGGACTGGGTTTCAGGAGGTTCGAATCCGC |
| KN_Contig_14906 | AGATTTACAAGTCAGCAATCCTCTTACATCAAGAGCAAGCTCAAGGAATC[T/C]GGCCTCACTCCCAGCGCCATCACCAACCTCAACGGAACAGTACAGCTGCG |
| KN_Contig_15007 | CCCTYGCCGGATCGGTACTTTCTCAACTTATATAGCTTGTCAACAATATA[T/G]CATTACTAGGAGCAGTTAATGTACCTATAGATTACAATGTTCTAAAAGTC |
| KN_Contig_15430 | CCTAACCAGTTCACTAGTGCAGTGGAGAGAAACTGGTCTGGACCCATTGG[T/C]CAGCTATCAAGTAATTTTCCAGAATCACAGAACGCTCAGTTTCAGCTTGC |
| KN_Contig_15467 | TATGAGACCGATCCCACTTGCTTCAATCATGGAGAAGCTGTCCCTCACAG[C/T]AGAGAACTATGGGAGCGTGCGCAAGTTCTACATTCAGACGCTGGACGACC |
| KN_Contig_15737 | CAGAATACTTCGGAAACCGTGTGAAGAATGTCATACTGAAATACAGTATA[G/C]AGAGACATTGGACATCTCTCAACGAGGAAACTGGTGGCATGAATGATGTT |
| KN_Contig_15945 | ATCATTTGAAGATGTCGGCAGAGGCAGCGGCGGCTGTTCCCAAGATTGCG[G/A]TACTCGGCGCCGGTATCTTTGTACGCACTCAGTACATTCCCAGGCTGAGG |
| KN_Contig_16506 | GAAAATTTAATGTACACTATTACTCCTGAAGAGATACCTTTCCAGCTTGG[C/G]TTGTCAAAAATGGATCTCAGGAAGACGTTGAAATCCAGCTTATCTGGAGT |
| KN_Contig_16523 | AAGGGAGGAATGCCTGGTAGTTTTTCTACTGCTGGCTACCAGGATTTCAG[G/A]TATGGCTATGATACCATGCAATCACCTATGTCGTGGTATGATGGCACATT |
| KN_Contig_16541 | AGATGCGATACGTCGTTGTTTTCTTGATTTTTACGCTTCTCGAGGCCACA[A/G]GATACTTCCGAGTTCGTCGCTCGTGCCTGACGATCCTACCGTGTTGCTTA |
| KN_Contig_16575 | CGAGCCCTCCCGACTTCGACAGATGAGCATCCGAAGATCTTTCGGATGAA[A/G]CTGTGGGCTACTAATGAAGTGAGGGCGAAGTCCAAATTCTGGTACTTTCT |
| KN_Contig_17527 | GATAAAAATATGCCAGGTGCAATTATGGCGATGGGGCGTCTTAATCGGCC[T/A]AGTATCATGATTTATGGTGGAACTATTAAGCCTGGTCATTTTGAGGGCAA |
| KN_Contig_18129 | GAGAACCTAAAGCACTTTGTGGAGGTCGTCTCCAAGCCTTGTGAGGAGGC[G/A]GCTCAGCGAAGAATTGACCGATTGTCGGGTGTTAAAGACGAGCTAGCAAA |
| KN_Contig_18374 | TTCTCTGGCGTCAGCCTAAACCCTCATAAGAGGAAGGGTTTGAAGACAGC[G/A]TCACTGAGCTTGGATCACTCGATTTGGTTCCACAGACCCGTAAAAGCTGA |
| KN_Contig_18721 | AAGAGATTAGAGAGCGATGGGCGCCTCCTCAGATCGTAGAGGCGAGGCCA[T/G]CCTTTAGGGTCGTGAATCGGAGGATCAGTAGGTCGCCGTTGGGGCGATCS |
| KN_Contig_19786 | CTTATTTGTACCTAACATTCGTCCTGAGGAAGAAGGGTCAAACAAATTCA[G/A]CTCGAAGGAACAACTCAAACAGAGGAAAGACCTCCATATCAGAGGATGAC |
| KN_Contig_20195 | GGGGGCCAATACAGTTCAAGTTTGCATCCCAAATGTACCCATCTCGTGGT[T/C]CAATGCTTTGGGGGACGAAAGTTCGAACATGCCTTGAAGCATGGATCAAG |
| KN_Contig_21375 | ATATTGRTCGGTGGGGGAGTCTGGGTTGAGATCGGAGTCGGAGTGGATCT[T/C]GGCGAGGATGTTATCGATCTCCTTGGAGACCTGCTCGGTGGGAGAATTGG |
| KN_Contig_22223 | TGAGCTTGCGAAGCTCAAGAAGCTGGCTAAGACTGAGGCGCTGGATGCTG[C/T]GCATATGGCTAAGGATGATGTGCTTCGATCTGAGAAACTTGATCAAATTC |
| KN_Contig_23151 | TGCAGCACTTGAAGCTGATCAGGCCAGAGAGCGCCAGAGGAGAGAGGAGC[A/T]GGAGCGTTTGGAACGAGAAGCTGCAGAAGCTGAAAGGAAGCTCAAGGAGG |
| KN_Contig_2345 | GCCCCAGCCAGCGGATTGGACAAGCCACGCATCGATCTTTCAGTGGAGCT[A/G]CAAATATTGGGTGTACAAGAATCCCTTGCAGGGCTGTGTGATTCTTTTAC |
| KN_Contig_23453 | GACCCCAACCAGAACACGGGCTTCTCCTTGCAATTCTGCAATGTCACTGC[A/G]GACTCCGACCTTGCGGCCTCGCCCAACAGTACGTACAGCTATCTTGGACG |
| KN_Contig_23660 | TTAGTTTTCCATTGGYCGAAAATTCATGACCGGCACACCTTCCTTCAACC[G/A]ACTGCTAGAGGGTTTGGTGGAGTTGTTAGGGCTGCTGTGACGGATATCCA |
| KN_Contig_23806 | GTTTCAGTTCCACACTATTACAACTTCCTGCTTGGAGTACGCCGAAGTGG[A/C]TTTGTAAAGAGTACCATGTCTTTACTGAGATCTCCAGATTCTGCTACTTC |
| KN_Contig_23808 | AACTGGATGAAATGCTGGAGAGTGTTGCACCAAATGCTTCCCTCTACAAT[G/A]CACTCATCCATGGATTTTGTTTGCGAGGCAAAAACCGCATTGGCAAAGAA |
| KN_Contig_24015 | TCACATTTTTCCCGTCATCCTGCTGCCGCTGGTACTTTTTTCACTGCAGT[T/A]CTCCTTGGGCTGAAGTTCTGCTCATGCCAGTCCCAGAGCAATCTGAAGAA |
| KN_Contig_24087 | TCACCCTCATGAATGGCCTTACYGCACCACTCAACATGAAGGTGTTCTAC[G/A]TCTCCTTCTCTGCTCACGCGGATTCTCTACAGTAACAAAGCGCATTTCTG |
| KN_Contig_24214 | GCGAGAGCAAGACAAGATCTGCCACAAGTGGTTAAGCTTGCAGAGAGTGG[C/T]GCCTTCAATCTTCAGAATACTGTTTCAAGAAAGTGCAAGTTTGAGGAAGC |
| KN_Contig_24233 | TTACTTCGTGGTGTCCGTCTTTTGCATGCACTGTCTGATCTGGCGTCTCG[T/C]CATGCTAGATTTGAGCAGATTCTGCTGGATGAAGTTAGGTATTCTGAACA |
| KN_Contig_24252 | TGACCCAGAAGCTGCGAGGAAAGATCCCTGCAAGTGCAGCAGTTCCACTC[C/A]TGTTCGACACAGGTAAAAGAACTAGCTTGTGACATGTGTAATCTTGAAAT |
| KN_Contig_24278 | CAGAAGAACCTTGCATTTGGTGCTGCAGCAAGCATGGTTCATCCAGCCAC[T/C]GGGTATTCTATTGGCCGCTCCTTATCAGAAGCTCCAAATTATGCATCTGT |
| KN_Contig_24424 | CATTGTCTGACACATTTTCATCCGAAAACTGCTACTCGTCTCCGTATGCT[G/T]ATCCAACTCTGTTGTAGATTGTTGAACATTCTTAGTGCCTCTAACCCCTT |
| KN_Contig_24540 | AAGTTCCCGCAGTTCTTCACCGGCAAGCGGAAGCCATGGAGAGCATTTCT[G/A]CTGTATGGTCCACCTGGAACTGGAAAATCATACTTGGCCAAGGCGGTAGC |
| KN_Contig_24564 | GGCGATTCAGGTCCAATTGTCACTGAAGCAGACATACAACATATTGTGTC[A/G]ACTTGGACTGGAATCCCAGTCGAGAAAGTATCGGTCGATGAGTCTGATCG |
| KN_Contig_24591 | ATTTGAGAAGATGATAATAAGTGAACTATTTTCAAATGTGGATATTAGAC[A/G]ACTGAATGCTCAAATGTGTACTGGAGAATCATGTCGATGTGGCAACAAAC |
| KN_Contig_24617 | TACTGTTTAATTTCCCTGTTGTTCCTGTGAAGCCTTAAGTTTCGATTGAT[C/T]GAGTGTTGAGCAGTTCTAGTCCGAGGTGATCGACAAACAAGAATTGCTCA |
| KN_Contig_24764 | AAAGCTGCTTATGACTTTGCTAGCGGTGAGCTCTTGAACCTTATTAAAGA[T/G]AAGTATGATCTCATTGGAAAGTTGCGGACTATGAAGCGCTATCTCCTTCT |
| KN_Contig_24786 | GAATCAAATGAGTTTGTTGGAGATAAAGTTGCATATGCACTCTCACAAGG[C/G]TTGAAGGTGATTGCTTGTGTTGGTGAGACCCTTGAGCAACGAGAATCTGG |
| KN_Contig_24813 | GGCAAAGATGATGTCATTAATGTAGGCGTAGCGGGTTGTTGCACTGGTCT[T/G]GCTCTGAGCTTTCCAGGTACACCCCAAGCTCTACTCCAGAGTTGTATCAC |
| KN_Contig_24874 | CGACTTCGGAAAGGATTAGATGAATCCTATTTGTGATTTTTGAGTGAACA[T/A]TATCTGGCATATATCCTGTGTCCTTTTACTTGCTGCTACGATGGTGTTGA |
| KN_Contig_24891 | CTTGCACAAGCATTCGACCAACTTGGTAACGACTGCCCTGATCTTGATGA[T/C]GTTCCATACCTGAAGAAGGTGTTTGAGACTGTTCAAGAGTTACTGTCAGA |
| KN_Contig_25004 | GCTAGATCGATACTCCTCTGTCGAAGAAGCCTATCATGACGGGAAACATC[G/A]CTTGGAAATGTTTCTCTCTAGGGTACACAAYGTTGATGTGCCGAGGCTGT |
| KN_Contig_25302 | GTTCTCAAATGTCAAGATTATCCTTCTGTCCGACGACTGCTTGATTCAAC[T/G]TCTTCCAAGAACACATCGACATGAGATCCATATCATATCATCAGTTGAAG |
| KN_Contig_25719 | GGTCCTCTTTCTGGAGACCGCGTGGCAGTTGCTCTATGGAATCGCTGTCC[C/T]GAGCCTGTCAAAATTACAGCTAAGTGGGAAGTGCTCGGCCTTGATACATC |
| KN_Contig_25878 | GAACTTCTTGGACCTGAGAAGGTGGATCCATCTGATGTGAAGCTTATCAA[G/A]GAAAAGCTTTTTGGCTACTCAACCTTTTGGGTAACAAGGGAGGAACCATT |
| KN_Contig_26066 | GCAACCAGCATGCTGAAGGGCTATCAGATTGGGGTTCTCGAAGGCAGTTA[T/C]GAGGCCGGTGGCAGCAGCGGCATCAATGGCACTGTGAAGAGCTACAAAAG |
| KN_Contig_26361 | GCTTGAAAAGGCTTTGAAGTCCGGCCAACTCCCAGCAGATCTTATGATTC[G/C]AGACAGTGATACTGCCATGTCTTCAGTCCAGGGCAAAGAAGACAGAATGG |
| KN_Contig_26393 | ATTGAGGGGCTGCCGAAGAAGTTCAGGGAGGGGGTGACGAAGGAGGAGGC[T/G]GAGGAGGCGAAGAAGCAGCTTGAGGGGGTTGGGCGAAGATTACCATTGTT |
| KN_Contig_26494 | ACGAGGTTGACCTTGTGGAACCTGTATCACATTTTATAGAGTCCGCTCGT[G/A]AAAATCTAGCCCCTAATGGTGATACAAATAAAGATGCTCACAAGGCCGTG |
| KN_Contig_26511 | ATGCTCTACACAGGCTCCACCAACGCGTCTGTCCAGGTGCAGAACCTAGC[T/C]TACCCAGAAGACCCCCGAAGATCCTCTTCTTCTGAAATGGGTGAAATCCG |
| KN_Contig_26918 | ATTGCATCATCAAAGACAACACTGACATCCACCAATCTGATGGACGAGAA[T/C]TCCTTCCAACAACCAAACAAGGTGGTACCCGTGAAGAACGTTCTTGGAAA |
| KN_Contig_29070 | AAGGTGTTTGTTCCATTAATGACTGCCTATGTTGTATGGCCAGTTGTTGG[C/T]TTACCAGCCTTGATCGCTGTCGTTCCATTTCTACTTGGATGCGCTGCACA |
| KN_Contig_31220 | GCCAAGTGCATACCTTCGTCTCACAAGACAAGTCTCACCCTCAGAGCAAC[G/A]AGGTGTACGAAATGTTGTATCAGATGGAATGGCAGCTCAGATTTGAAGGC |
| KN_Contig_35884 | TTAAGGATGACAGCCGCCGTTAGAGTGTTACCATGCACTACTCGCACCTT[C/A]AGATCCGGGTGTTTGCTCACAAAGAGTGTTCCTCCTCCATTCAACGCTTC |
| KN_Contig_35911 | CAGCCTCTGAATATTAGCCTGAAGGCCCTCCAATGAACGGTTCCTGCGTC[T/G]GTGATCAACAGAAATACCAATGGTTGGTGCAAGCTTTCTCGGAATGCCCG |
| KN_Contig_35995 | TTACTACGAAGCTTCGACAGAAGCGAGTCCAAGCTATCCAGTCTGGTTCC[A/G]AGAATCGTAATCTTCCTCCGAATYGCAGATGTATGGCGTATGGCTTCTGG |
| KN_Contig_36144 | TGTAGCTTTGCTACCAATGTAAGTCCAAATAGATTTAAAAATATGTAAGC[G/A]GGATTAAGTATCTGACCCATAGCAGCACCATTAACTAAGATAGAACTAAT |
| KN_Contig_36373 | ATGATACTGCAGATGGACAAGAATATGATGAAGATATCAAAGGTCAGGGC[G/A]GTCATGGCTTGGTCACTGAGCTTCATGTTCTTCCCTCCTAGCTTGTCGAC |
| KN_Contig_36745 | GTGGTGCTCATATCAAATCCACCATCTAAAAGCTCTCCACTGTCAGAGTA[C/A]GTTGGCTTTGCCATACTTGCCAATTGTTTATAGGAAAACCAGTAAGCCAT |
| KN_Contig_37759 | AGAACGCTTTGACTTGGGCCAAAGAATCATGCCAATAAGGTCCGCACCAG[T/C]TTGTGCAGCCATTTCTGCATCTTTAGCAGTAGTGACACCACACATCTTCA |
| KN_Contig_4728 | GCAAGTTCTACCACCGCTTCATCGATCCCGATTTTGTGGTTGAGACAATG[T/A]CTTCAAGCAGCTCCTCTCAGCCACCTAGAGCATCAACAAGCTCTTCATCA |
| KN_Contig_5028 | CCATCGAAGGCTCTACTTGCAGTGAGTGGTCGAATGCGTGAGCTTCAAAG[C/T]GAGCATCACTTGAAGGCATTTGGTATACAGGTTTCATCTGCTGGTTATGA |
| KN_Contig_5107 | GTCCTGAAGCTCAGGAAGGTGGTCCGATCGGCCTTATTCAGAATGGAGAT[A/G]TGATCACCATTGATGTTCGTAAGAAAACAATTGATGTTGCATTGACTGAG |
| KN_Contig_5253 | CGGCGGGAGTGGGGAATTACTTCCAATGCAGTGAAGAAAGGGGGACTTTA[T/C]ACATTTGACCTTGGTTCACAGTACAAGTACAAGAACACTATTGTGGATCT |
| KN_Contig_5399 | GCCTGCATGCGATCGATACGTTGGACCCTGATATGGTCATTGGACAAGAG[T/G]GTGTTCGTTATTATCATGACATGATACTGGAAATGATCAAGTGGGGTTAC |
| KN_Contig_5464 | ATAAGGTTAGAGGAGGAGAATTCGAGGAATAGGGAGATGGTTCGGGAGGT[T/A]TATGAGAGGGCGATTGGGAATGTTCCTCCAGAACCTAAGAAGCGGTACTG |
| KN_Contig_6119 | GCAGGAAGTGAAGAACACSTAGCTGCTTGCAAGAAGCTTGGTGCTGATGT[C/A]TGCATAAATTACAAGACTGAGGACTTTGCTCTGCGGGTTAAGGAAGAGAC |
| KN_Contig_6306 | CTACTGTCCTCTGCGATGATGTTGAGACATCTACAATTCCCTTCATTTGC[A/T]GATCGGCTAGAAACTGCTGTCAAGGGGGTGATTGAAGAAGGCAAATATAG |
| KN_Contig_6543 | AAGCTAAGTAAGTGGGCAGAAGCATACGGTGGTTTCTTTAAACCGTCGTC[A/G]TACTTGGAAGAAAGGGCCAAAAAAGGGTGTGTCATTGAGCACACCAAATT |
| KN_Contig_655 | GCCAAGGTAGATGCTGTGGTGTACTTGGTGGACTCTGCTGACAAGGAGAG[A/G]TTTGCAGAGTCGAAGAAGGAGCTGGATGCACTCCTCTCAGACGATTCACT |
| KN_Contig_6738 | TGCGCCATTGGGTCCGGTGCACAGGACATGGCGATGGCCGATGAGCCTAA[A/G]CGAGGGACACTTGAAGCTAAGAAGAAGTATGCTCCAGTGTGCGTCACCAT |
| KN_Contig_7249 | ATCCATCATTGGAGGTTATGCCTACAGGTCGACGACTGATCCATGCATGT[C/T]TGGAAGGTATTTGTATGCGGACCTGTATGCTGAAGCAATGTGGGCAGGCA |
